# GDF-15 Predicts Epithelioid Hemangioendothelioma Aggressiveness and Is Downregulated by Sirolimus through ATF4/ATF5 Suppression

**DOI:** 10.1158/1078-0432.CCR-23-3991

**Published:** 2024-09-16

**Authors:** Silvia Stacchiotti, Silvia Martini, Sandro Pasquali, Anna M. Frezza, Alessia Beretta, Stefano Percio, Mara Lecchi, Monica Tortoreto, Marta Barisella, Paola Collini, Gian Paolo Dagrada, Alessandra Merlini, Paul H. Huang, Andrew Jenks, Robin L. Jones, William D. Tap, Matilde Ingrosso, Carlo Morosi, Silvia Brich, Claudia Giani, Paolo Verderio, Paolo G. Casali, Hugh Leonard, Alessandro Gronchi, Valentina Zuco, Nadia Zaffaroni

**Affiliations:** 1 Medical Oncology Unit 2, Cancer Medicine Department, Fondazione Istituto di Ricovero e Cura a Carattere Scientifico (IRCCS) Istituto Nazionale Tumori, Milan, Italy.; 2 Molecular Pharmacology Unit, Department of Experimental Oncology, Fondazione IRCCS Istituto Nazionale dei Tumori, Milano, Italy.; 3 Unit of Bioinformatics and Biostatistics, Department Epidemiology and Data Science, Fondazione IRCCS Istituto Nazionale dei Tumori, Milan, Italy.; 4 Pathology Unit, ASST Fatebenefratelli Sacco, Milan, Italy.; 5 Soft Tissue Tumor Pathology Unit, Department of Advanced Diagnostics, Fondazione IRCCS Istituto Nazionale dei Tumori, Milano, Italy.; 6 Department of Oncology, University of Turin, Turin, Italy.; 7 Division of Molecular Pathology, Institute of Cancer Research, London, United Kingdom.; 8 The Institute of Cancer Research, London, United Kingdom.; 9 The Royal Marsden NHS Foundation Trust, London, United Kingdom.; 10 Department of Medicine, Memorial Sloan Kettering Cancer Center, New York, New York.; 11 Department of Radiology, Fondazione IRCCS Istituto Nazionale dei Tumori, Milano, Italy.; 12 Chair of Trustees of the EHE Rare Cancer Charity UK, Charity Number 1162472, Kingston-Upon-Thames, United Kingdom.; 13 Sarcoma Service, Department of Surgery, Fondazione IRCCS Istituto Nazionale dei Tumori, Milano, Italy.

## Abstract

**Purpose:**

Epithelioid hemangioendothelioma (EHE), an ultra-rare sarcoma, poses therapeutic challenges because of limited efficacy of conventional chemotherapy in advanced cases, necessitating exploration of new treatment avenues and identification of novel aggressive biomarkers. This study aimed at (i) utilizing a patient-derived xenograft model of EHE and its associated cell line to assess the efficacy of sirolimus and (ii) analyzing two distinct patient cohorts to pinpoint circulating biomarkers of EHE aggressiveness.

**Experimental Design:**

A patient-derived xenograft model and corresponding cell line were established from a patient with advanced EHE, demonstrating consistency with the original tumor in terms of histomorphology, *WWTR1::CAMTA1* fusion presence, and genomic and transcriptomic profiles. Two independent patient series were employed to investigate the association between growth/differentiation factor 15 (GDF-15) serum levels and EHE aggressiveness.

**Results:**

ELISA analyses on EHE cell culture medium and blood from EHE-carrying mice revealed the release of GDF-15 by EHE cells. Sirolimus exhibited markedly higher antitumor activity compared with doxorubicin, concurrently reducing GDF-15 expression/release both *in vivo* and *in vitro*. This reduction was attributed to the drug-induced inhibition of phosphorylation/activation of 4E-BP1 and subsequent downregulation of the GDF-15 transcription factors ATF4 and ATF5. Blood sample analyses from two independent patient series showed a significant correlation between GDF-15 and EHE aggressiveness.

**Conclusions:**

This study identifies GDF-15 as a novel biomarker of EHE aggressiveness and underscores the superior efficacy of sirolimus compared with doxorubicin in our experimental models. The observed inhibition of GDF-15 release by sirolimus suggests its potential as a biomarker for monitoring the drug’s activity in patients.

**Translational Relevance**

This study exploited a patient-derived xenograft and the paired cell line of epithelioid hemangioendothelioma (EHE), a YAP/TAZ-driven ultrarare sarcoma, and found that EHE cells produce and release growth/differentiation factor 15 (GDF-15), a member of the TGFβ superfamily. Results indicate that the assessment of circulating GDF-15 holds promise of refining risk stratification of patients with EHE. Additionally, the evidence that sirolimus downregulates the expression/release of GDF-15 in EHE models suggests the possibility for the cytokine to be used for defining sirolimus effectiveness and detecting disease progression in patients with EHE. Moreover, the patient-derived xenograft we established in this study from a patient with EHE characterized by high-risk features and that maintains the molecular profile of the originating clinical sample represents a valuable model to generate preclinical findings and hypotheses to be translated to the clinic with the aim to improve the management of higher risk patients.

## Introduction

Epithelioid hemangioendothelioma (EHE) is an ultrarare and translocated vascular sarcoma that exhibits endothelial differentiation (1–4). In 90% of EHE cases, a fusion involving the WW domain-containing transcription regulator 1 (*WWTR1*)/transcriptional coactivator with a PDZ-motif (*TAZ*) and the calmodulin-binding transcription activator 1 (*CAMTA1*) genes has been identified (5), whereas approximately 10% of tumors display a fusion between yes-associated protein (*YAP*) and transcription factor E3 (*TFE3*) genes, known as YAP-TFE3 (1). These fusion proteins WWTR1(TAZ)CAMTA1 and YAP-TFE3 drive a unique transcriptome by simultaneously hyperactivating a TEA domain (TEAD)–dependent transcriptional program and modulating the chromatin environment through interactions with the Ada2a-containing histone acetyltransferase (ATAC) complex (6). Forced activation of YAP/TAZ is believed to be the driving factor in EHE (7, 8). Additionally, secondary genomic alterations characterize approximately half of EHE, including mutations in CDKN2A/b, RB, APC, ATRX, XRCC2, and FANCA (9, 10).

Patients with EHE lack tumor biomarkers to refine their prognostic risk stratification and effective tumor-specific treatment options, particularly for patients with highly aggressive disease. Indeed, more than half of all patients are diagnosed with metastatic disease and the behavior of EHE can display significant variability, spanning from a relatively indolent course to an exceptionally aggressive one. Indicators that suggest a less-favorable prognosis, include the extent of disease, involvement of serosal membranes and the presence of paraneoplastic signs and symptoms, such as pleural effusions, fever, weight loss, asthenia, and severe tumor-related pain (4, 11). Conventional anthracycline-based chemotherapy and other systemic agents available for the treatment of advanced sarcomas have marginal activity in EHE (3, 12, 13). The highest clinical activity has been reported so far, but only retrospectively, for the mTOR inhibitor sirolimus (3, 13, 14).

Progress in the understanding of EHE biology and identification of new therapeutic targets have been limited, beyond disease rarity, by the lack of patient-derived models that recapitulate the genomic complexity of this tumor beyond genetically engineered mouse models (GEMM; refs. 7, 8).

Growth and differentiation factor 15 (GDF-15) is a member of the TGFβ superfamily and is mainly found in the placenta and prostate under normal conditions, although it is induced by various stress signals (15–17). Elevated GDF-15 levels are associated with inflammatory conditions, myocardial ischemia, autoimmune diseases, and various cancer types (17). In cancers, elevated GDF-15 levels are often associated with tumor aggressiveness and poor outcome (18–21). In sarcomas, GDF-15 expression is elevated in metastatic osteosarcoma, linked to reduced survival, and promotes cell migration through the TGF-β pathway (22). Additionally, GDF-15 may serve as a serum biomarker to distinguish uterine sarcomas from leiomyomas (23).

Utilizing a recently established patient-derived xenograft (PDX) model of EHE and its corresponding cell line obtained from a patient with an aggressive form of EHE characterized by severe inflammatory symptoms, which is to the best of our knowledge the first patient-derived model of this disease, our research demonstrates that EHE cells produce and secrete GDF-15. Furthermore, the mTOR inhibitor sirolimus modulates the secretion of GDF-15 by downregulating ATF4 and ATF5 transcription factors. In patients, GDF-15 serves as a predictive marker for the aggressiveness of EHE and holds implications for monitoring the effectiveness of sirolimus as a treatment option for patients with EHE, especially in the more aggressive cases marked by serosal involvement and symptom deterioration.

## Materials and Methods

### Development of PDX and PDX-derived cell line

The EHE PDX and the corresponding cell line were derived from an aggressive form of EHE with an unbalanced *WWTR1::CAMTA1* translocation. A detailed description of the clinical tumor is reported in Supplementary Material S1.

The EHE PDX was generated from fresh EHE specimen collected immediately after surgical resection from the previously described patient. Clinical tumor was aseptically dissected, cut into ∼3-mm^3^ fragments and incubated in Matrigel at 4°C for 30 minutes (Cultrex Basement Membrane Extract, Type 3, Pathclear, Bio-Techne srl). At least five fragments were grafted subcutaneously into the right flank of 6-week-old female CB17/lcr-Prkdcscid (SCID, RRID: IMSR_ARC:SCID) mice (Charles River Laboratories, RRID: SCR_003792). Tumor growth was followed by biweekly measurement of tumor diameters with a Vernier caliper, and tumor weight (TW) was calculated according to the formula TW = *d*2 × *D*/2, in which *d* and *D* represent the shortest and the longest diameters, respectively. After the third passage in mice, the PDX was considered established. Detailed description for PDX generation and evaluation of tumor growth are reported elsewhere (24, 25).

SCID mice were maintained in a pathogen-free facility where temperature and humidity were kept constant and had free access to food and water. Mice’s weight was routinely monitored during and after drug treatment for the whole experiment.

The use of patient material to generate the PDX model was approved by the institutional ethical committee (INT-139/17 and INT-174/20) and the patient consented. Generation of PDX and *in vivo* drug experiments were approved by the Institutional Animal Care and Use Committee and authorized by the Italian Ministry of Health (project approval code: 234/2018-PR and 104/2023-PR) in compliance with international policies and guidelines (Italian implementation of Directive 2010/63/EU on the protection of animals used for scientific purposes). Detailed description for PDX generation and evaluation of tumor growth are reported elsewhere (24, 25) and detailed in Supplementary Material S1.

The EHE cell line was generated in our laboratory. On April 2021, the PDX at the fifth passage was cut into pieces of ∼ 1 mm^3^ and enzymatically digested with collagenase (200 U/mL; Collagenase from *Clostridium histolyticum*, Merck, RRID: SCR_001287) in free-serum DMEM/F-12 for 3 hours at 37°C. Cells were resuspended in DMEM/F-12 supplemented with 10% fetal bovine serum (DMEM/F-12 complete medium) to inactivate collagenase and applied to a cell strainer (100 µm, Corning), centrifuged at 500 × *g* for 5 minutes, and resuspended in DMEM/F-12 complete medium. Tumor cells were then propagated in DMEM/F-12 complete medium and maintained in an incubator at 37°C and 5% CO_2_. This cell line was established after 10 passages (July 2021) based on its constant growth pattern and doubling time (25 ± 3 hours). At this passage, we performed single tandem repeat analysis by the AmpFISTR Identifiler PCR Amplification Kit (Applied Biosystems, RRID: SCR_005039) to authenticate this cell line. The established cell line was not contaminated by murine cells as indicated by the assessment of species-specific beta-2-microglobulin through RT-PCR. Cells were periodically tested for *Mycoplasma* (MycoAlert Mycoplasma Detection Kit, LT07-318, Lonza, RRID: SCR_000377).

The cell-based experiments reported in the article have been carried out at passages 11 to 20. The authentication of PDX and its corresponding cell line was performed through microsatellite analysis by the AmpFISTR Identifiler PCR amplification kit (Applied Biosystems, RRID: SCR_005039).

The EHE cell line was characterized for the presence *WWTR1(TAZ)-CAMTA1* fusion by FISH analysis. The cell line was tumorigenic and able to generate palpable tumors 1 week after injection of 5 × 10^6^ cells into the flanks of SCID mice.

### PDX characterization

The consistency of the EHE PDX with the originating clinical tumor was assessed in terms of histomorphology, presence of the *WWTR1::CAMTA1* fusion, and genomic and transcriptomic profiles. Doubling time of EHE PDX is around 26 days (Supplementary Fig. S1).

#### Histopathologic analysis

Four-micrometer sections of formalin-fixed, paraffin-embedded (FFPE) tumor tissue obtained from the patient’s surgical specimen and sacrificed mice were stained with hematoxylin and eosin for morphological evaluation and Ki67 (1:400, Ab Ki67, Clone Mib1, Agilent, Cat. #GA626, RRID: AB_2687921). IHC analysis was performed at room temperature on the Dako Autostainer Link 48 AS480 (Agilent, RRID: SCR_013575), as previously described (26). The IHC slides were scanned at 10× magnification. The digital images of Ki67 expression were analyzed using the open-source DIA software QuPath software v. 0.1.2 (RRID: SCR_018257). The Ki67 labeling index was expressed as number of Ki67-positive nuclei/overall number of nuclei × 100.

Pathologists with expertise in soft-tissue sarcomas compared morphologic features of human and PDX tumors.

#### FISH analysis

FISH was performed on 2-µm FFPE tumor sections with a commercially available probe for *TFE3* (ZytoLight SPEC *TFE3* break-apart probe; ZytoVision) and in-house labeled bacterial artificial chromosome (BAC) clones for *WWTR1* (RP11-941L15 and RP11-1151O19) and *CAMTA1* (RP11-1114C18; RP11-1120I14, RP11-338N10, and RP11-60J11). The BAC clones were obtained from the Children’s Hospital Oakland Research Institute BACPAC Resource Center and labeled to produce a WWTR1 dual-color break-apart probe and a *WWTR1::CAMTA1* dual-color dual fusion probe.

A commercially available *MET*/centromere seven dual-color probe and a dual-color *WT1*(11p11.3)/*FLI1*(11q24.3) in the house-made BAC probe were used to validate differentially expressed copy-number variations (CNV) in tumor relative to PDX.

#### Genomic profile

DNA was isolated from FFPE EHE clinical and PDX samples using the GeneRead DNA FFPE kit (Qiagen, RRID: SCR_008539) following the manufacturer’s instructions. DNA was quantified using a Qubit dsDNA HS Assay Kit (Thermo Fisher Scientific Inc., RRID: SCR_008452), and DNA quality was assessed by TapeStation 4200 (Agilent 4200 TapeStation System, RRID: SCR_018435). The OncoScan CNV Assay (Thermo Fisher Scientific Inc.) was implemented following the manufacturer’s instructions to detect genome-wide copy-number gains and losses, loss of heterozygosity, including copy neutral loss of heterozygosity, and a panel of somatic mutations. Data were visualized with the karyoploteR tool implemented into R.

#### Transcriptomic profile

Total RNA was extracted from frozen EHE clinical and PDX samples with an RNeasy Mini Kit (Qiagen). The concentration and the purity of the RNA starting material were measured on a spectrophotometer, and RNA integrity number was measured on an Agilent TapeStation 4200 (Agilent 4200 TapeStation System, RRID: SCR_018435, Agilent Technologies). cDNA libraries were synthesized from 200 ng total RNA with an RNA Prep Kit with Tagmentation (Illumina, Agilent Technologies, RRID: SCR_013575) and quantified by Qubit Assay (Thermo Fisher Qubit 2.0 Fluorometer Qubit 2.0, RRID: SCR_020553). After the quality control performed with the fastQC tool (RRID: SCR_014583), sequences were aligned using STAR (RRID: SCR_004463) against the human reference genome hg38. Read counts were determined according to the protocol used for library preparation. Raw data were normalized using the trimmed mean of Mvalue, according to the edgeR (RRID: SCR_012802) package of the R environment, and filtered, discarding reads under the 10th percentile of expression variance across samples and lacking an associated official gene symbol. Reads mapping on the same gene symbol were collapsed, summing their counts. The gene expression matrix and the preprocessing pipeline were deposited at Gene Expression Omnibus (accession number GSE246152, RRID: SCR_005012). The correlation between the gene expression profiles of PDX and clinical tumor was evaluated with the Spearman coefficient in order to detect a possible nonlinear relationship.

### Drug activity studies

For *in vitro* studies, doxorubicin (Adriblastina, RRID: SCR_025575, Accord Healthcare) was diluted in sterile saline solution before use. Sirolimus (Rapamycin, MedChemExpress), was dissolved and diluted in DMSO (0.5% final concentration in culture medium). Exponentially growing cells were treated, the day after seeding, with increasing drug concentrations for 6 days. The antiproliferative activity was evaluated by cell counting using a Coulter counter (Z Series Coulter Counter, Beckman Coulter GmbH, RRID: SCR_008940). Drug concentrations able to inhibit cell proliferation by 50% (IC_50_) were calculated from dose–response curves. Cells treated with solvent (DMSO at 0.5% final concentration in culture medium) was used as control.

For *in vivo* studies, when tumor burden reached about 100 to 120 mm^3^, SCID mice were randomized to receive different treatments. Each experimental group consisted of nine mice; six mice were used to assess the drug effect on tumor growth over time, whereas three mice were sacrificed after the end of treatment to collect blood from mice, to carry out hematoxylin and eosin, Ki67, and Western blot analysis.

Mice were treated with doxorubicin (Adriblastina, Accord Healthcare, RRID: SCR_025575) after dilution in saline solution. Sirolimus (Rapamycin, MedChemExpress, DBA) was dissolved in aqueous vehicle solution containing 2% ethanol, 10% of PEG-400, and Tween-80 solution 1:1 (v/v). Drugs were administered at dosages and schedules as reported in Supplementary Table S1. Mice treated with vehicle solution were used as control.

Each TW measure was normalized to the TW of the same mouse at the start of treatment (RTW), and treatment efficacy was evaluated in the normalized TW curve of individual mice as TW inhibition percentage (TWI%) at nadir.

### Small interfering RNA–mediated gene knockdown

Human GDF-15 small interfering RNA [siRNA; Silencer Select Pre-designed siRNA ID: 18258 (#2); ID: 18259 (#1) and 228510 (#3)] was purchased from Thermo Fisher Scientific Inc., whereas human ATF4 [ON-TARGETplus SMART pool siRNA (468) ID: L-008822-00-0005] and human ATF5 [ON-TARGETplus SMART pool siRNA (22809) ID: L-005125-00-0005] were purchased from Dharmacon (Horizon Discovery, Carlo Erba Reagents Srl).

One day after seeding EHE cells were exposed to each siRNA (33 nmol/L) for 24 hours in free-serum DMEM/F-12 using Lipofectamine RNAiMAX (Thermo Fischer Scientific Inc.) as a transfecting agent. A control siRNA (Silencer Select Negative Control #2 siRNA, Thermo Fisher Scientific Inc. or On-TARGET plus Non-targeting Pool, Dharmacon) with no homology to any known human mRNA was used as transfection control. Twenty-four hours after siRNA transfection, cells were maintained either in complete medium for different intervals and then assessed for the expression of siRNA target genes as well as for their proliferative potential or in serum-free medium for the assessment of GDF-15 release.

To investigate the role of GDF-15 in tumor growth, EHE cells were transfected with GDF-15 or negative control siRNA according to the protocol described above. Twenty-four hours after the end of transfection, 5 × 10^6^ cells/SCID mouse were injected subcutaneously in 100 μL of serum-free medium and Matrigel (Cultrex Basement Membrane Extract, Type 3; 1:2 ratio).

### Transcriptomic profile of EHE cells after siRNA-mediated GDF-15 knockdown

Total RNA extracted at different time intervals from cells transfected with GDF-15 siRNA or control siRNA was processed according to standard NEBNext Ultra II Directional RNA Library Prep Kit (New England Biolabs, RRID: SCR_013517) for Illumina protocol, and mRNA was selected with oligo-dT beads starting from 800 ng of total RNA. Libraries were quantified and quality checked on an Agilent 4200 TapeStation system (High Sensitivity D5000, Agilent Technologies), and then sequenced on an Illumina NovaSeq platform (Illumina NovaSeq 6000 Sequencing System, RRID: SCR_016387) with paired-end reads 150 bp long, with a depth of 27 million cluster/sample.

Differential expression analysis was performed by removing data heteroscedasticity with the voom method implemented into the edgeR package. A linear model was employed to evaluate differentially expressed genes using the limma package (LIMMA, RRID: SCR_010943; ref. 27), measuring both the fold change (FC), logarithmically (base 2) transformed, and the t-statistic. Taking into account multiple comparisons to assess significant changes, a threshold of 0.05 was considered for the FDR *P* value correction. Gene set enrichment analysis (GSEA) was performed on the hallmark collection of the Molecular Signatures Database, and genes were ranked according to the t-statistic as implemented into the GSEA package (RRID: SCR_003199). An FDR threshold of 0.05 was applied to assess significant enrichments.

### Western blot analysis

Lysates were obtained from exponentially growing cells and from frozen xenotransplants after pulverization by the Mikro-Dismembrator II (B. Brown Biotech International).Equal amounts of proteins were separated by SDS-PAGE, transferred onto nitrocellulose membranes, and incubated with the following primary antibodies: anti–GDF-15 (Santa Cruz Biotechnology, Cat. # sc-377195, RRID: AB_2895563), anti–phospho-mTOR (Ser2448, Santa Cruz Biotechnology, Cat. # sc-293133, RRID: AB_2861149), anti-mTOR (Santa Cruz Biotechnology, Cat. # sc-517464, RRID: AB_3186240), anti–phospho-p70 (Thr389, Cell Signaling Technology, Cat. # 9205, RRID: AB_330944), anti-p70 (Cell Signaling Technology, Cat. # 9202, RRID: AB_331676), anti–phospho-4E-BP1 (Ser65, Cell Signaling Technology, Cat. # 9451, RRID: AB_330947), anti–4E-BP1 (Cell Signaling Technology, Cat. # 9644, RRID: AB_2097841), anti-ATF4 (Abcam, Cat. # ab184909, RRID: AB_2819059), anti-ATF5 (Abcam, Cat. #ab184923, RRID: AB_2800462), and anti-vinculin (Sigma-Aldrich, Cat. # V9131, RRID: AB_477629). Horseradish peroxidase–linked goat anti-rabbit IgG (Cell Signaling Technology, Cat. # 7074, RRID: AB_2099233) or horseradish peroxidase–linked horse anti-mouse IgG (Cell Signaling Technology, Cat. # 7076, RRID: AB_330924) antibodies were used to detect primary antibodies. Immunoreactive bands were revealed by the enhanced chemiluminescence detection system ECL (GE Healthcare, RRID: SCR_000004).

The membranes were cut to allow simultaneous incubation of primary antibodies detecting proteins with different molecular weights on the same membrane. Membranes were stripped and reincubated with different primary antibodies and successively with the specific peroxidase secondary antibodies. For the preparation of figures, we cropped original film Western blot images to generate a unique panel with different proteins. Molecular massed were detected using the Precision Plus Protein Standard (Bio-Rad Laboratories, RRID: SCR_008426). Quantification of band intensities for each blot of in the article is reported in Supplementary Tables S1–S3.

### Proteome Profiler Human XL Cytokine Array

The Proteome Profiler Human XL Cytokine Array Kit (ARY022B, R&D Systems Inc., RRID: SCR_006140) was used to screen plasma of patients with EHE and healthy donors, as well as serum-free medium of EHE cells, for a large panel of secreted proteins. Spots array were revealed by the enhanced chemiluminescence detection system ECL (GE Healthcare). Chemiluminescence spots were analyzed to find the first evidence of saturation on the reference spot.

### ELISA

Levels of human GDF-15 were measured by using a Quantikine ELISA Kit (R and D Systems Cat# DGD150, RRID:AB_2877710) in plasma samples collected from patients with EHE and healthy donors as well as in plasma from of mice carrying the EHE xenotransplants and in the serum-free medium of EHE cells. Mouse blood (approximately 300–500 µL/mouse) was collected under deep terminal anesthesia by cardiac puncture. For comparative purposes, plasma from mice carrying pleomorphic sarcoma xenografts as well as serum-free culture medium of pleomorphic sarcoma, dedifferentiated sarcoma, angiosarcoma, and normal endothelial cell (HUVEC, RRID: CVCL_2959) lines were analyzed. The optical density of the wells was determined with a microplate reader (Bio-Rad iMark Microplate Absorbance Reader, RRID: SCR_023799, Bio-Rad) set to 450 nm. GDF-15 concentrations were measured in duplicate samples. The values from each assay were extrapolated from a standard curve fit. As plasma samples and conditioned medium were diluted, the concentration read from the standard curve was multiplied for dilution factor to obtain the final pg/mL concentration.

### RT-qPCR

Total RNA was extracted with the miRNeasy Mini kit (Qiagen) from cells transfected with gene-specific siRNAs.

One microgram total RNA was reverse-transcribed using High-Capacity cDNA Reverse Transcription Kit (4368814, Thermo Fisher Scientific Inc.). Gene expression levels were assessed by RT-qPCR using specific TaqMan assays (GDF-15: Hs.PT.58.40089589, Thermo Fisher Scientific Inc.; ATF4: Hs.PT.56a.744435.g; and ATF5: Hs.PT.58.25040399, Integrated Technology Enterprise Inc., RRID: SCR_012186). Amplifications were run in the QuantStudio 12K Flex Real-Time PCR System (Thermo Fisher Scientific Inc.). GAPDH was used as housekeeping control gene (Hs.PT.39a.22214836 Thermo Fisher Scientific Inc.). Data were reported as relative quantity (RQ = 2^−ΔΔCt^): being ΔCt the difference between the threshold cycle (Ct) of the target gene and the Ct of the housekeeping gene and ΔΔCt the difference between ΔCt of the sample and ΔCt of the calibrator. The calibrator corresponded to the sample transfected with control (NEG) siRNA.

### Migration and invasion

Cell migratory capability was assessed through the wound healing assay by analyzing cell ability to close a gap in the culture monolayer. Six days after siRNA transfection, EHE cells were seeded in two well silicone inserts with a defined cell-free gap (80209, ibidi). Twenty-four hours after seeding, inserts were removed and fresh complete medium was added. Images were captured by phase-contrast microscopy immediately after insert removal (*t*_0_) and 24 hours later (*t*_24_ hours).

The invasion ability of cells was assessed using the Transwell permeable supports (8.0 μm Polycarbonate Membrane, 6.5 mm Insert; #3422, Corning) coated with Matrigel (#356231, Corning). Six days after siRNA transfection, EHE cells were seeded in the upper chamber in free-serum medium, whereas the lower chamber was filled with of complete medium. Twenty-four hours after seeding, cells that passed through the microporous membrane invading the Matrigel were fixed to the filter with 100% ethanol, stained with 0.4% sulforhodamine B (Merck) in 1% acetic acid, and washed. Images of five random fields per chamber were captured by phase-contrast microscopy and quantified using the ImageJ 1.47q software (RRID: SCR_003070).

### Patient cohorts and blood collection

Two series of patients with EHE were analyzed, the retrospective (training cohort) and the prospective (testing cohort) ones.

The retrospective cohort included 20 patients with a diagnosis of EHE who had a consultation at the Department of Cancer Medicine of Fondazione IRCCS Istituto Nazionale dei Tumori (INT) and had their blood sampled (April 2019 to January 2020). Blood samples were also collected from 32 healthy patients (21 female/11 male) with a median age of 35 years (range 21–57).

The prospective study included 21 consecutive adult patients with centrally reviewed and molecularly confirmed pathologic diagnosis of EHE seen at INT and Royal Marsden Cancer Centre, London, UK eligible for an international, observational prospective study on EHE (INT174/20). Detailed information on this clinical trial as well as the criteria used to define lower and higher risk patients are reported in Supplementary Material S1. All procedures performed were in accordance with the ethical standards of the institutional and/or national research committee and with the 1964 Helsinki Declaration and its later amendments. Blood samples were also collected from an additional 32 healthy patients (17 female/15 male) with a median age of 49 years (range 20–58). All patients enrolled in the two cohorts gave their written informed consent to participate in this collection of data and samples.

Whole blood samples were collected into sterile BD Vacutainer K2EDTA tubes (Becton Dickinson), centrifuged, aliquoted plasma, and stored at −80°C until use.

### Statistical analysis

In the retrospective patient series, a single blood sample was available to assess the level of the GDF-15. In the clinical trial (prospective cohort), the GDF-15 expression level used for the analysis corresponded to (i) the last measurement, if the patient was always been at low risk; (ii) the first measurement, if the patient was always been at high risk; and (iii) the first measurement at high risk, if the patient switched from low to high risk during the follow-up.

The analyses were performed within each cohort, because of different recruitment periods, and overall at the end of the process. GDF-15 expression levels were compared between healthy donors and patients. Subsequently, higher risk patients were separated from lower-risk patients, in order to study GDF-15 according to tumor risk. Wilcoxon nonparametric test and two-tailed *t* test were used for the within cohort analyses and the overall analysis, respectively. An internal validation was performed by using the bootstrap method because of the small sample size in the comparison between higher and lower risk patients. Only resamples with at least 10 high-risk patients were included in the procedure (28).

The statistical analysis of differences between recorded variables in the performed experiments was performed with GraphPad Prism software version 9.4 (GraphPad Software Inc., RRID: SCR_002798). The *P* values were calculated using two-tailed paired *t* test or one-way ANOVA and Tukey *post hoc* test. Comparisons reaching *P* < 0.05 were considered statistically significant.

### Data availability

Transcriptomic data of EHE tumor and the paired PDX as well of EHE cell line transfected with GDF-15 or control siRNA were deposited at Gene Expression Omnibus (accession number GSE246152). All other relevant data generated during the current study are available from the corresponding author on reasonable request.

## Results

### GDF-15 was released by tumor cells in patient-derived models of EHE

**Figure 1.**
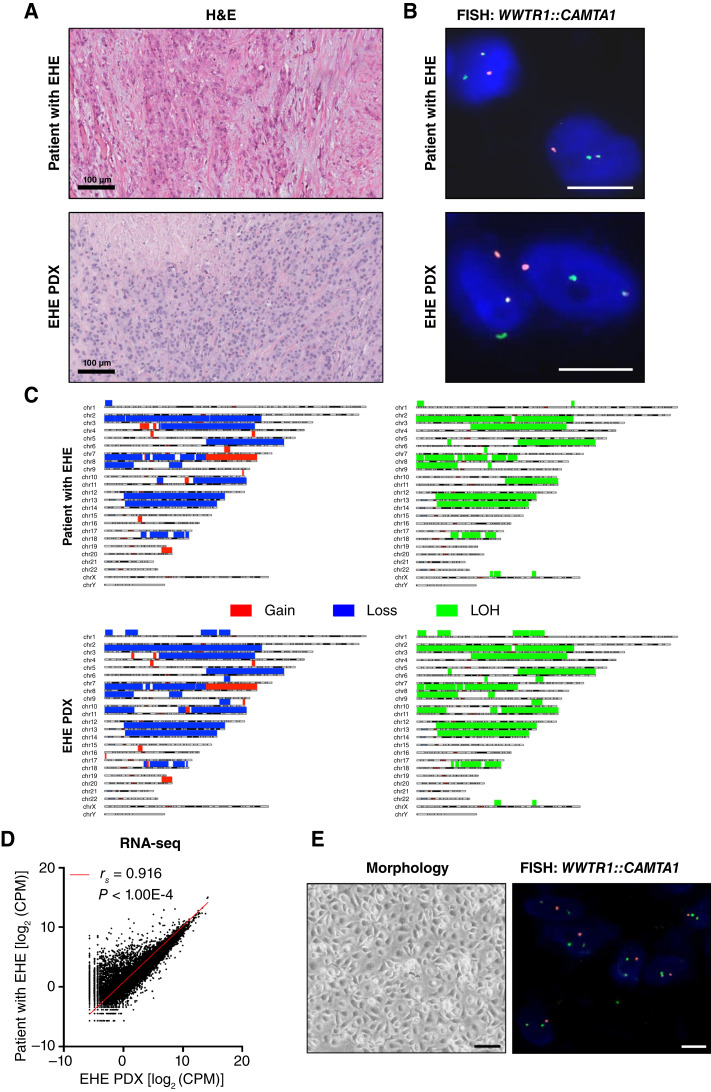
Characterization of EHE PDX and paired cell line. Representative pictures of the EHE clinical sample and corresponding EHE PDX model. **A,** The histology was assessed on hematoxylin and eosin–stained slides. Scale bar, 100 µm. *WWTR1::CAMTA1* dual-color dual-fusion FISH pattern in diploid tumor cells showing one single fusion, consistently with the genomic profile. **B,** Genomic profile of EHE clinical tumor (left) and PDX (right). **C,** Loss (blue) and gain (red) were depicted in the top. LOH (green) was reported in the bottom (scale bar, 10 µm). **D,** Scatter plot of significant correlation between the transcriptome of the clinical tumor and the paired PDX (Spearman correlation, *r*_*s*_ = 0.916; *P* < 0.001). **E,** Morphology of the EHE cell line derived from the PDX growing as monolayer (left, scale bar, 250 µm) and confirmation of *WWTR1::CAMTA1* translocation at FISH analysis (right, scale bar, 10 µm). H&E, hematoxylin and eosin; LOH, loss of heterozygosity; RNA-seq, RNA sequencing.

GDF-15 is known to be released by a variety of cell types (29), including tumor cells of different histologies (15–17). To assess whether EHE cells produce and secrete GDF-15, a human EHE PDX model was generated together with its derived cell line. This PDX properly recapitulated the histologic and molecular findings of the originating clinical tumor. Specifically, both tumors were characterized by the presence of a solid growth pattern, focal marked cytologic atypia, and areas with abundant spindle cells or necrosis (Fig. 1A). FISH analysis revealed the presence of the pathognomonic unbalanced *WWTR1::CAMTA1* unbalanced translocation (Fig. 1B). Genomic analysis of the clinical EHE and PDX revealed similar profiles with gain and loss in several chromosomes (Fig. 1C). Both clinical tumor and PDX were characterized by *CDKN2A* loss, one of the most common secondary genomic alterations in EHE (Supplementary Fig. S2; refs. 7, 8). Interestingly, in both the tumor and the PDX, the first hit is represented by a large ch9p deletion (Fig. 1C), involving the 9.p21 locus that includes the *CDKN2A* gene, whereas the second hit in the tumor is represented by a further genomic deletion (135 Kb deletion) leading to homozygous loss. In the PDX, the second hit is represented by the pathologic mutation c.238C>T p.Arg80Ter. However, some differences in the chromosomal architecture of the PDX compared with the clinical sample were observed mainly in chromosomes 1, 7, and 11. For instance, the 7q31.1q31.31 region was duplicated in the clinical tumor and deleted in the PDX. This was confirmed by FISH analysis, showing a tandem duplication of *MET *in the clinical tumor and the loss of the duplicated region in the xenograft that leads to the CNV profile observed (Supplementary Fig. S3A). Again, the 11p/11q copy-number profile indicated that the CNV ratio 11p/11q was unbalanced in the clinical tumor, with a relative loss of 11q, and balanced in the PDX. This was confirmed by FISH using a dual-color *WT1*(11p11.3)/*FLI1*(11q24.3) probe (Supplementary Fig. S3B).

RNA sequencing revealed a highly significant correlation of the transcriptomic profiles of the clinical tumor and the PDX (*r*_*s*_ = 0.916; *P* < 0.001; Fig. 1D). The gene expression profiles of both PDX and clinical sample clustered together with those of EHE clinical samples analyzed in two publicly available datasets (Supplementary Fig. S4A). Consistent with the clinical tumor, the EHE PDX expressed high levels of ERG and CD31 (PECAM1) transcripts (Supplementary Fig. S4B). However, GSEA (RRID: SCR_003199) revealed differences between the PDX and clinical tumor in some gene sets related to angiogenesis, epithelial–mesenchymal transition and KRAS-up signaling (Supplementary Fig. S4C).

The translocation of *WWTR1::CAMTA1* was also confirmed in the PDX-derived EHE cell line by FISH analysis (Fig. 1E).

**Figure 2.**
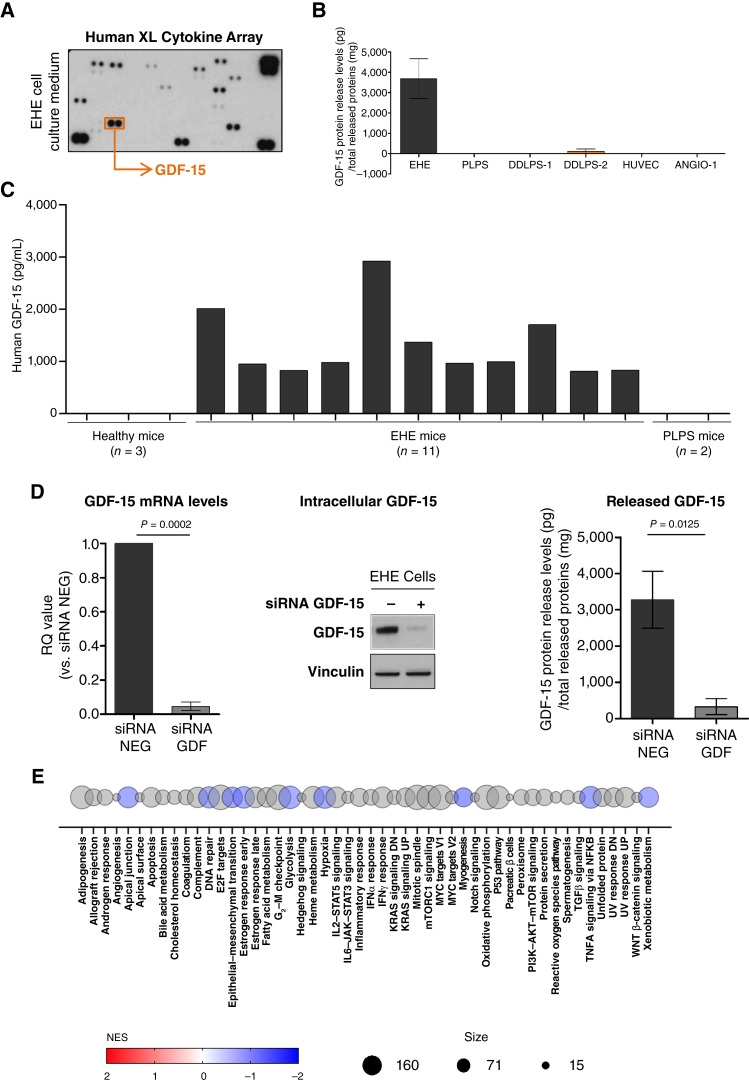
GDF-15 was released by tumor cells in patient-derived models of EHE. **A,** Assessment of released cytokines in the culture medium of EHE cells using the Human XL Cytokine Array. Detection of GDF-15 by ELISA in culture medium of EHE cell lines and cell lines of PLPS and DDLPS-1 and -2. **B,** Data were normalized as amount (pg) of released GDF-15 to total released (mg) proteins. RT-qPCR and ELISA results are reported as mean ± SD of three independent experiments. **C,** Detection of GDF-15 by ELISA in the plasma of healthy mice and mice carrying EHE PDX or PLPS PDX. **D,** siRNA-mediated downregulation of GDF-15 in the EHE cell line as detected at the mRNA level by RT-qPCR (left), protein level by Western blotting (middle), and as cytokine released in cell culture medium by ELISA 3 days after transfection (right). ELISA data were normalized as amount (pg) of released GDF-15 to total released (mg) proteins and reported as mean ± SD of three independent experiments. **E,** GSEA (RRID: SCR_003199) was employed on Hallmark (H) collection of the Molecular Signature Database, showing a limited number of modulated pathways following GDF-15 knockdown. DDLPS, dedifferentiated liposarcoma; PLPS, pleomorphic liposarcoma.

Using the Human XL Cytokine Array on EHE cell serum-free medium, we found the presence of GDF-15 among the different released cytokines (Fig. 2A). The quantitative analysis of EHE cell culture medium by ELISA confirmed the presence of released GDF-15 (Fig. 2B). Analysis performed in the culture medium of primary cell lines we generated from patients with soft-tissue sarcoma histologies other than EHE, such as pleomorphic liposarcoma, dedifferentiated liposarcoma, or angiosarcoma, as well as of a normal endothelial cell line (HUVEC) showed a lack or a negligible level of released GDF-15 (Fig. 2B). Consistently with *in vitro* results, the presence of released GDF-15 was observed to a variable extent in the blood of a panel of mice carrying EHE xenotransplants of different size but not in healthy mice and in those carrying pleomorphic liposarcoma xenotransplants (Fig. 2C).

To investigate a possible role for GDF-15 in EHE cells, we transiently downregulated its expression by a siRNA (#2), which was preliminarily identified as the most efficient in downregulating GDF-15 expression among three different siRNAs (Supplementary Fig. S5A–S5C). At 3 days after transfection, an almost complete abrogation of GDF-15 was observed at both mRNA and protein level (Fig. 2D; Supplementary Table S1), together with its markedly reduced release in cell culture medium (Fig. 2D). Such a reduced GDF-15 expression and release was still appreciable, although to a lesser extent, 10 days after transfection (Supplementary Fig. S6A–S6C). RNA sequencing carried out on EHE cells transfected with either GDF-15 siRNA or control siRNA (NEG) did not reveal a profound modification of the transcriptomic profile following GDF-15 knockdown (Supplementary Fig. S7), with only a few modulated, mainly downregulated, pathways as showed by GSEA (Fig. 2E; RRID: SCR_003199). Consistently, GDF-15 silencing did not impair the proliferative, migration, and invasion potential of EHE cells (Supplementary Fig. S8A–S8C). Moreover, GDF-15 knockdown did not modify the ability of EHE cells to growth as tumor xenografts in mice (Supplementary Fig. S8D).

### Sirolimus downmodulated GDF-15 release in patient-derived models of EHE

**Figure 3.**
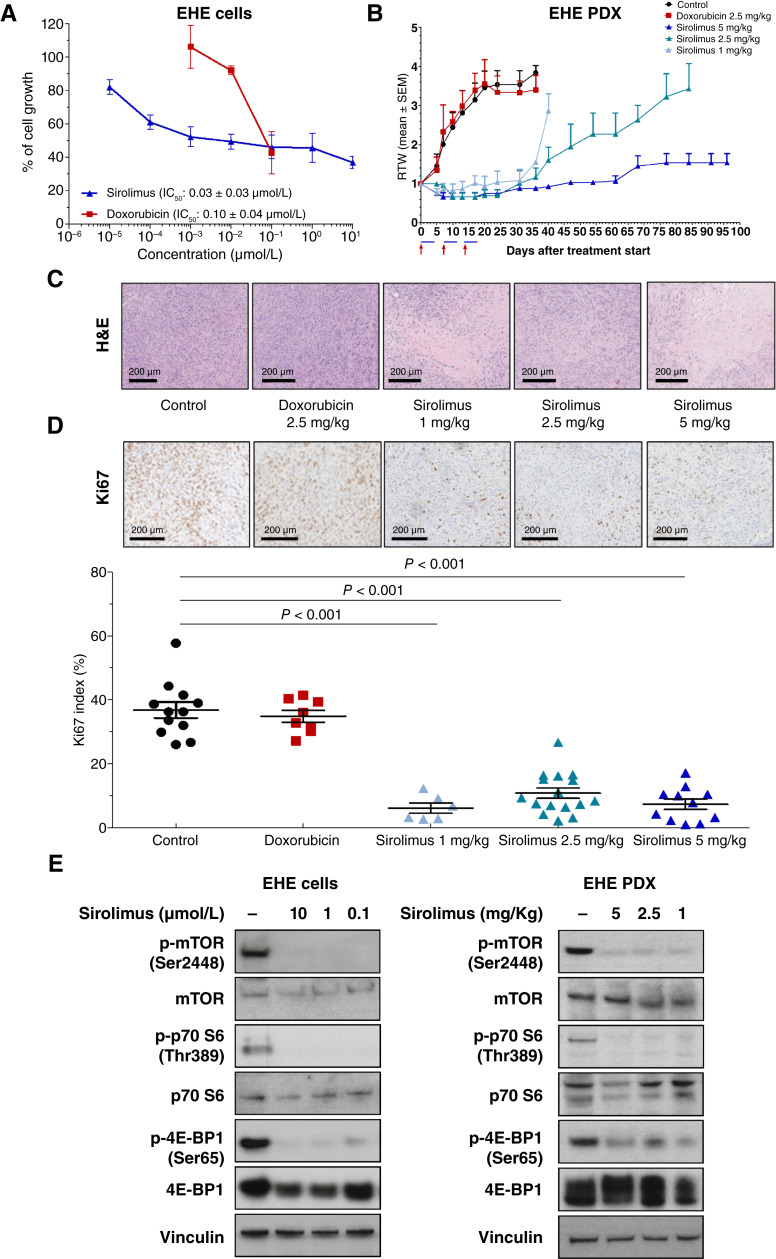
Sirolimus inhibited the growth of patient-derived models of EHE. **A,** Cell growth inhibition curves obtained after exposure to different doses of doxorubicin or sirolimus. Data are reported as the percentage of drug-treated cells compared with control cells and represent mean ± SD of three independent experiments. **B,** Growth curves reporting the RTW (mean ± SEM) in control and doxorubicin- or sirolimus-treated mouse groups (nine mice/group), in which 1 indicates the tumor weight at the beginning of the treatment. The arrows indicate when drugs were administered. **C,** Histomorphologic evaluation of tumors obtained from untreated and drug-treated mice. **D,** Ki67 immunostaining of tumors obtained from untreated and drug-treated mice (top) and quantification of Ki67 index (bottom). Symbols reported in the represent counted fields. Histomorphologic analysis and Ki67 immunostaining were performed on tumors excised from mice at the end of drug treatment. Scale bar, 100 μm. Data are reported as means ± SD of three independent experiments. **E,** Western blot analysis of downstream mTOR pathway in untreated cells and cells treated with different sirolimus concentrations for 3 days (left) and in tumors removed from untreated and sirolimus-treated mice at the end of treatment with different drug doses (right). Cropped images of selected proteins are shown. RTW, relative tumor weight.

Based on the notion that GDF-15 is a mitokine induced in the context of an integrated mitochondrial stress response driven by the mTOR complex 1 (mTORC1; ref. 30) and that the mTOR inhibitor sirolimus is the best treatment option for patients with EHE requiring systemic treatment (3, 14), we assessed the effect of sirolimus in our EHE models. In the EHE cell line, sirolimus was more active that doxorubicin, the standard first-line chemotherapeutic agent for soft-tissue sarcomas, as indicated by the lower concentration able to inhibit cell growth by 50% (IC_50_: 0.03 vs. 0.10 µmol/L; Fig. 3A). In the EHE PDX model, doxorubicin showed a negligible activity, whereas sirolimus induced a maximum tumor weight inhibition (mTWI) of 68% to 81% as a function of its dose (Fig. 3B; Supplementary Table S4). Tumors started to regrow after the end of treatment with 1 and 2.5 mg/kg sirolimus, whereas 5 mg/kg sirolimus resulted in a delayed tumor regrowth (Fig. 3B). The histomorphologic analysis of tumors excised from drug-treated and untreated mice showed that tumors treated with sirolimus had post treatment changes, such as sclerojalinosis together with tumor necrosis (Fig. 3C; Supplementary Fig. S9). The latter characteristic seems likely tumor-related as it could be detected both in controls and tumors treated with doxorubicin. The proliferation rate, as detected by Ki67 index in the same tumors, indicated a marked reduction (*P* < 0.001) in the percentage of proliferating cells after treatment with sirolimus but not doxorubicin (Fig. 3D). Western blot carried out on EHE cells and xenografts confirmed the downregulation of the mTOR downstream signaling pathway after sirolimus treatment (Fig. 3E; Supplementary Table S2).

**Figure 4.**
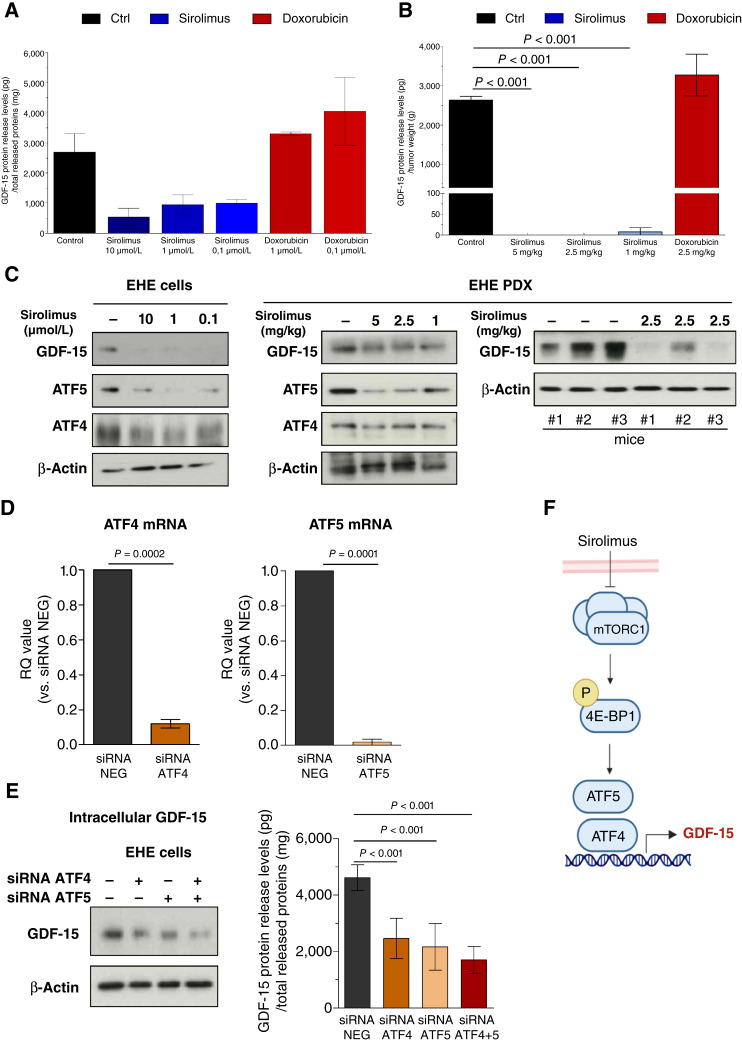
Sirolimus downmodulated GDF-15 release in patient-derived models of EHE through the inhibition of ATF4 and ATF5. **A,** GDF-15 released in culture medium of control cells and cells exposed for 3 days to doxorubicin or different sirolimus concentrations as detected by ELISA. Data were normalized as amount (pg) of released GDF-15 to total released (mg) proteins and reported as mean ± SD of three independent experiments. **B,** GDF-15 released in the blood collected from untreated mice and after the end of treatment with doxorubicin or different sirolimus doses. Data were normalized as amount (pg) of released GDF-15 to tumor weight (g) and reported as mean ± SD of three mice/experimental group. **C,** Western blot analysis of GDF-15, ATF4, and ATF5 expression in untreated cells (−) and cells treated with different sirolimus concentrations for 3 days (left) and in tumors removed from untreated (−) and sirolimus-treated mice after the first round of treatment with different drug doses (right). Western blot data of GDF-15 expression obtained on tumors from three untreated mice (−) and three mice exposed to 2.5 mg/kg sirolimus. Cropped images of selected proteins are shown. **D,** siRNA-mediated downregulation of ATF4 (left) and ATF5 (right) in the EHE cell line as detected at the mRNA level by RT-qPCR. **E,** Effects of ATF4 and ATF5 downregulation on GDF-15 protein expression as detected by level by Western blotting (left) and on cytokine release in cell culture medium as measured by ELISA (right). ELISA data were normalized as amount (pg) of released GDF-15 to total released (mg) proteins and reported as mean ± SD of three independent experiments. Cropped images of selected proteins are shown. **F,** The working model of sirolimus-induced GDF-15 downregulation. Sirolimus-induced mTORC1 inhibition led to reduced 4E-BP1 phosphorylation/activation followed by decreased ATF4 and ATF5 expression, thus resulting in GDF-15 downregulation. (**F,** Created with BioRender.com.)

We then assessed whether sirolimus affected release of GDF-15 from EHE cells. Three-day exposure to sirolimus caused a marked, although nonstatistically significant, reduction of GDF-15 released in the culture medium of EHE cells (Fig. 4A). Interestingly, an almost complete abrogation (*P* < 0.001) of the levels of circulating GDF-15 was consistently observed in the blood of mice after treatment with different doses of sirolimus (Fig. 4B). These effects were observed after normalization for total released proteins for EHE cells or tumor weight for EHE PDX, suggesting a direct effect of sirolimus on released GDF-15 (Fig. 4A and B). Conversely, doxorubicin did not appreciably modify the release of GDF-15 by EHE cell both *in vitro* (Fig. 4A) and *in vivo* (Fig. 4B). Sirolimus treatment reduced GDF-15 mRNA in EHE cells (Supplementary Fig. S10A), resulting in a marked reduction of its protein levels both in EHE cells and PDX (Fig. 4C; Supplementary Table S3).

### GDF-15 downmodulation by sirolimus was mediated by inhibition of ATF4 and ATF5

Based on previous evidence showing that mTORC1 controls ATF4 expression by regulating the translation and stability of its mRNA through 4E-BPs (31) and that GDF-15 is a direct target of ATF4 (32), we evaluated whether the inhibition of 4E-BP1 phosphorylation/activation induced by sirolimus eventually affected the expression of ATF4. Western blot carried out on EHE cells exposed to different sirolimus concentrations and in tumors excised from mice treated with different sirolimus doses consistently showed a reduction in the expression of ATF4 (Fig. 4C; Supplementary Table S3). A reduced expression of ATF5 was concomitantly observed (Fig. 4C; Supplementary Table S3).

As a previous report indicated that RNAi-mediated downregulation of ATF5 in hepatocellular carcinoma cells caused a reduced expression of GDF-15 (33), we next validated both ATF4 and ATF5 as regulators of GDF-15 in EHE cells through their siRNA-mediated knockdown. Indeed, in presence of a marked downregulation of ATF4 (*P* = 0.0002) and ATF5 (*P* = 0.0001) expression (Fig. 4D), each transcription factor was able to reduce the abundance of GDF-15 in EHE cells (Fig. 4E; Supplementary Table S3) as well as its release in the culture medium (Fig. 4E). This inhibitory effect on GDF-15 expression and release was slightly enhanced following concomitant ATF4 and ATF5 knockdown. A possible model for sirolimus-induced GDF-15 downregulation is depicted in Fig. 4F. Conversely to what observed in GDF-15 downregulated cells (Supplementary Fig. S8A), siRNA-mediated ATF4/ATF5 silencing reduced EHE cell growth (Supplementary Fig. S10B), possibly as a consequence of reduced expression of ATF4/5 target genes other than GDF-15. Moreover, we evaluated the GDF-15 expression and release after the concomitant effect of sirolimus and ATF4/ATF5 knockdown (Supplementary Fig. S10C and S10D), and no significant differences in the extent of GDF-15 decrease were observed.

### Circulating GDF-15 levels correlated with disease aggressiveness in patients with EHE

**Figure 5.**
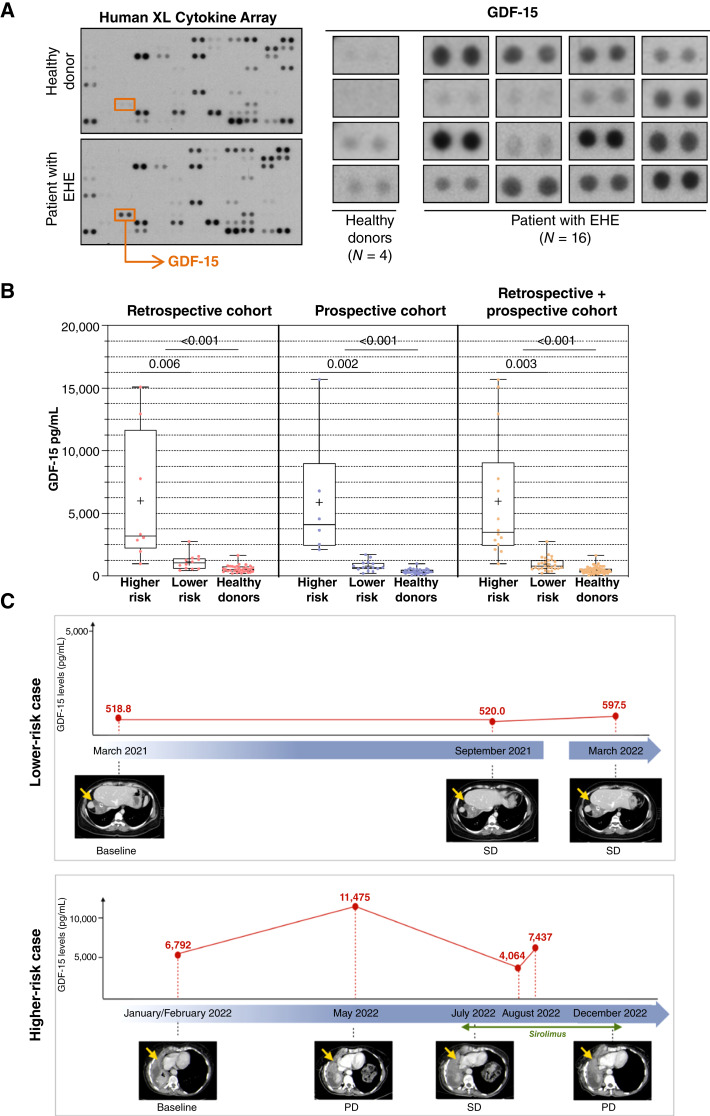
Circulating GDF-15 levels correlated with disease aggressiveness in patients with EHE. **A,** Analysis of circulating cytokines in plasma samples of 16 patients with EHE and four healthy donors using the Human XL Cytokine Array showed higher GDF-15 levels in patients compared with healthy donors. **B,** GDF-15 levels, as detected by ELISA and expressed in pg/mL plasma, in patients with EHE compared with healthy donors in a training (retrospective; 8 higher-risk and 12 lower-risk patients and 32 healthy donors) and a testing (prospective; 6 higher-risk and 15 lower-risk patients and 32 healthy donors) cohort and in both the cohorts. **C,** Two clinical cases are reported showing levels of GDF-15 in a lower-risk patient (top) with stable disease at follow-up and a higher-risk patient (bottom) who had an initial tumor response and levels of GDF-15 reduced after being started on the mTOR inhibitor sirolimus followed by an increase in GDF-15 before the CT scan that showed disease progression. PD, progressive disease; SD, stable disease.

To assess the potential clinical relevance of GDF-15 for EHE, we initially carried out an exploratory qualitative analysis of circulating cytokines using the Human XL Cytokine Array on plasma samples of patients with EHE compared with healthy donors (Fig. 5A). On the basis of this finding, we assessed using a specific ELISA assay the amount of circulating GDF-15 in plasma samples in two cohorts. The former included a series of healthy individuals (*N* = 32) and a retrospective series of patients with EHE (*N* = 20), which comprises those previously assessed with the cytokine array, whereas the latter included a new series of healthy individuals (*N* = 32) and a prospective series of patients with EHE within a clinical observational study (*N* = 21). Table 1 reports the main demographic and clinicopathologic characteristics of patients in the two cohorts. No appreciable differences were observed among the patient cohorts, with the only exception of a higher prevalence of bone and soft-tissue lesions in the prospective cohort. No association was observed between GDF-15 levels and age or sex in both patient and healthy donors.

**Table 1.** Demographic and clinicopathologic characteristics of patients with EHE in the two study cohorts.

Characteristic	Retrospective cohort (*N* = 20)	Prospective cohort (*N* = 21)
Sex (*N*, %)		
Male	5 (25)	6 (29)
Female	15 (75)	15 (71)
Lower-risk disease (*N*, %)	12 (60)	15 (71)
Higher-risk disease (*N*, %)	8 (40)	6 (29)
Age at diagnosis (median, IQR)	46.5 (36–51)	49 (43–57)
Presentation (*N*, %)		
Unifocal	0 (0)	3 (14)
Systemic	20 (100)	18 (86)
Organ involvement (*N*, %)		
Lung	14 (70)	15 (71)
Liver	13 (65)	13 (62)
Bone	4 (20)	11 (52)
Soft tissue	2 (10)	5 (24)
Pleura	7 (35)	2 (10)
Others	4 (20)	5 (24)
Serosal involvement (*N*, %)		
Absent	14 (70)	17 (81)
Present	6 (30)	4 (19)
Effusion (*N*, %)		
Absent	13 (65)	16 (76)
Present	7 (35)	5 (24)
Symptoms (*N*, %)		
Absent	12 (60)	14 (67)
Present	8 (40)	7 (33)

Results showed a statistically significant higher expression of GDF-15 in patients with EHE compared with healthy donors in both retrospective (*P* < 0.001) and prospective (*P* < 0.001) cohorts (Fig. 5B). Patients with higher-risk EHE did show the highest levels of GDF-15 in both cohorts (retrospective cohort, *P* = 0.006; prospective cohort, *P* = 0.002; Fig. 5B), highlighting the association of GDF-15 levels with EHE aggressiveness. By analyzing the two cohorts together in order to increase the sample size, the statistically significant association between GDF-15 levels with EHE (*P* < 0.001) and disease aggressiveness (*P* < 0.03) was confirmed (Fig. 5B). The internal validation procedure, based on 89,970 bootstrap resampling, was applied on the overall analysis and the robustness of the aggressiveness results was confirmed (*P* < 0.001).

To further support the value of GDF-15 level as a biomarker of EHE aggressiveness, we reported on a higher-risk patient (Fig. 5C), who developed disease progression at the right lung together with pleural effusion between February and May 2022, which was associated to a marked increase of GDF-15 level from 6,792 to 11,475 pg/mL. This patient was started on the mTOR inhibitor sirolimus, which resulted in disease stability and a reduced level of GDF-15 (4,064 pg/mL). This patient had a follow-up in August 2022, when the level of GDF-15 was markedly increased (7,437 pg/mL). A contrast-enhanced CT scan performed in December 2022 demonstrated EHE progression in the right lung. A patient with a lower risk EHE (Fig. 5C), presenting with distant metastasis sited in the lungs, liver, and bone, which remained stable at follow-up, without systemic symptoms maintained the level of GDF-15 stable between March 2021 (518.8 pg/mL) and March 2022 (597.5 pg/mL).

## Discussion

This study exploited *in vitro* and *in vivo* patient-derived models of EHE to reveal that EHE cells secrete GDF-15 and demonstrated that the expression and secretion of GDF-15 are suppressed by the mTOR inhibitor sirolimus, achieved through the downregulation of ATF4 and ATF5 transcription factors. We posited that GDF-15 levels might correlate with the aggressiveness of EHE, and we were able to establish that circulating GDF-15 serves as a novel biomarker for assessing aggressiveness in patients with EHE exploiting two case series, including an ongoing prospective clinical study.

GDF-15 is a divergent member of the TGFβ superfamily (15–17). Under physiologic conditions, GDF-15 expression is mostly limited to the placenta and prostate (34), and being a stress response cytokine (30), its expression is induced by different stress signals (35–37). Elevated levels of GDF-15 are present in different human pathologic conditions, including inflammation, myocardial ischemia, and different cancer histologies (16, 17), being often associated with tumor aggressiveness (15, 38, 39). Interestingly, GDF-15 was found to exert immune-regulatory functions in cancer and noncancer pathologic conditions (40). Importantly, its documented ability to promote tumor immune escape has pointed out its possible relevance as a novel targetable immune checkpoint to be exploited for cancer immunotherapy (15).

GDF-15 molecular regulation and downstream signaling are largely unknown. Multiple independent studies have reported that GDFN family receptor α–like (GFRAL) acts as a receptor for GDF-15 (41, 42). Following binding, the GDF-15–GFRAL complex was found to interact with the RET proto-oncogene and promote the oncogenic signaling via ERK and AKT pathways (41, 42). As the expression of GFRAL is highly restricted to neurons in the area postrema and nucleus tractus solitarius of the brainstem where it is responsible for GDF-15–mediated anorexia (43, 44), GFRAL-independent effects exerted by GDF-15 in the different pathologic conditions might be mediated by other yet unidentified receptors (45).

GDF-15 was shown to be expressed/released by tumor cells of different histological origin (15–17) and, in the context of tumor microenvironment, by tumor-associated macrophages and fibroblasts (46, 47). Very limited information is available with regard to GDF-15 expression in sarcomas. The expression of this cytokine was found to be upregulated in metastatic compared with nonmetastatic osteosarcoma and patients with high serum GDF-15 levels exhibited a decreased survival (22). In addition, GDF-15 was found to promote cell migration and invasion in osteosarcoma cell lines by sustaining the TGFβ pathway (22). Furthermore, based on higher serum levels of GDF-15 found in patients with uterine sarcomas compared with those with leiomyomas, circulating GDF-15 was proposed as novel biomarker to discriminate between the two diseases (23).

This study showed the association between circulating GDF-15 levels and EHE aggressiveness in two independent cohorts of patients with lower- or higher-risk disease, including a prospective clinical study. Currently, tumor features such as mitotic activity, grading, size, and the type of genomic fusion (*WWTR1::CAMTA1* vs. *YAP::TFE3*) as well as tumor-related patient symptoms (i.e., paraneoplastic), including weight loss, pain, cough, hemoptysis, and clinical signs such as anemia and pleural effusions, are exploited to identify patients at higher risk of a disease progression and worse prognosis. Some patients are classified as having lower risk disease and go on to develop new symptoms being then upstaged as higher risk. Additionally, variation of GDF-15 levels after starting a patient on sirolimus may further assist clinicians in understanding the effectiveness of sirolimus, information that is particularly relevant for patients at high risk of rapid disease progression and the associated challenges of radiological response evaluation in EHE. Early results presented here will be investigated in the ongoing prospective study with mature data.

To assess whether EHE cells express and release GDF-15, thus contributing to the circulating GDF-15 in patients with EHE, we generated, to the best of our knowledge, the first EHE PDX model and the derived cell line. This PDX represents an unprecedented source of information for EHE as it retains the main histomorphologic features as well as genomic and transcriptomic profiles of its paired clinical tumor, although some differences in the architecture of specific chromosomes as well as in the in the enrichment of specific gene sets have been observed. Indeed, genomic differences between the original clinical tumor and PDX are expected, and this may be due to several issues, including clonal selection in PDX coupled with the lack of immune surveillance in the SCID mouse as already discussed in the literature (48) This model, which was established from a patient with higher-risk EHE, diverges from the two published GEMM of EHE (7, 8), which only rely on the oncogenic properties of the *WWTR1::CAMTA1* translocation, as this PDX displays greater genomic complexity and carries additional secondary molecular alterations, such *CDKN2A* loss, strongly influencing tumor behavior. Indeed, loss of *CDKN2A* in one EHE GEMM was found to cooperate with the *WWTR1::CAMTA1* fusion to enhance tumor progression (10, 49).

Results generated on our *in vivo* and *in vitro* models provided evidence that EHE cells express and release GDF-15. However, differently from experimental models of other tumor histotypes showing a direct effect of GDF-15 on tumor phenotype (50), siRNA-mediated knockdown of GDF-15 failed to affect proliferative, migratory, and invasive potential of EHE cells. This is consistent with a previous report indicating that GDF-15 silencing in melanoma cells did not influence tumor cell behavior *in vitro* but inhibited the tumor growth in immunocompetent mice by suppressing angiogenesis (51). Conversely, in EHE cells GDF-15 knockdown failed to affect tumor take and growth kinetics in SCID mice possibly because we worked with human tumor cells in the context of the murine microenvironment of immunodeficient mice (48).

Based on currently available literature, the role of GDF-15 on tumor onset and progression is far from being elucidated. In this context, existing evidence supports both tumor-suppressive and tumor-supportive roles of GDF-15, as a function of cancer type and stage (15–17). Specifically, GDF-15 seems to limit tumor growth in early tumor stages, whereas cancer cells may exploit GDF-15 to escape immune surveillance in later stages, promoting a protumorigenic environment which supports tumor proliferation and metastatic spread (15–17).

Following previous reports indicating a modulatory effect of some anticancer drugs on GDF-15 expression (50), we assessed the effects of sirolimus (which is currently considered the best treatment option for patients with EHE) and doxorubicin (the standard first-line chemotherapeutic agent for soft-tissue sarcomas) in our EHE models. Results obtained on the NCI-60 cell lines upon 15 drug treatments indicated that DNA damaging agents induce GDF-15 expression more effectively that drugs with different mechanisms of action and that the extent of induction was context dependent (50). Consistently with clinical evidence, a superior antitumor activity of sirolimus compared with doxorubicin was observed in the EHE PDX, confirming the ability of PDX models to properly reproduce clinical results with regard to the activity of drugs, which we had previously observed in PDX models generated from other soft-tissue sarcoma histologies (52, 53). Sirolimus markedly inhibited the expression of GDF-15 and its release in the blood of EHE PDX carrying mice as well as in the culture medium of EHE cells, whereas doxorubicin did not appreciably modify GDF-15 release from EHE cells and xenografts. However, because a more pronounced inhibitory effect was observed at the protein level compared with mRNA, it is plausible that could modify the expression of other factors involved in *GDF*-*15* gene posttranscriptional regulation. Looking for the mechanisms responsible for such downregulation, we found that sirolimus-induced inhibition of 4E-BP1 phosphorylation led to a reduced abundance of ATF4, and its expression is known to be controlled by mTORC1 by regulating the translation and stability of its mRNA through 4E-BPs (31). siRNA-mediated ATF4 inhibition resulted in reduced expression of GDF-15, which is a direct target of ATF4 and presents ATF4-binding consensus sequences located between exons 1 and 2 (30). We also found a reduced expression of ATF5 after sirolimus exposure in our EHE models, which likely contributed to GDF-15 inhibition, in accordance with a previous report indicating that RNAi-mediated downregulation of ATF5 caused a reduced expression of GDF-15 in hepatocellular carcinoma cells (33) and also confirmed in our EHE cell line. However, based on the results of *in vitro* and *in vivo* experiments we cannot rule out the possibility that other factors are responsible for GDF-15 expression besides ATF4/5.

Overall, results from our study indicate that the assessment of circulating GDF-15 holds promise of refining risk stratification of patients with EHE and suggest the possibility for the cytokine to be used for defining the effectiveness of sirolimus and detecting disease progression. The observational clinical study ongoing at our institutions includes the longitudinal monitoring of GDF-15 in patients diagnosed with EHE and will help confirming the clinical utility of GDF-15. Moreover, the PDX we established in this study from a patient with EHE characterized by high-risk features, and that maintains the molecular alterations of the originating clinical sample, represents a valuable model to generate preclinical findings and hypotheses to be translated to the clinic with aim to improve the management of higher-risk patients. This represents an advantage compared with GEMM, which provided invaluable information about EHE onset (7, 8) but due to the lack of secondary alterations, likely limit their predictive clinical value to lower-risk EHE.

## Supplementary Material

Supplementary Material 1 Supplementary Material 1. Patient and human tumor characterization and prospective study.

Supplementary Figure 1 Supplementary Figure 1. Representative growth curves of the EHE PDX at different passages in mice.

Supplementary Figure 2 Supplementary Figure 2. CDKN2A loss in EHE clinical tumor and PDX.

Supplementary Figure 3 Supplementary Figure 3. Chromosomal architecture of the PDX compared to the clinical sample in chromosomes 7 and 11.

Supplementary Figure 4 Supplementary Figure 4. Transcriptomic profiles of EHE clinical tumor and PDX.

Supplementary Figure 5 Supplementary Figure 5. Effect of different siRNA-mediated down-regulation of GDF-15 in EHE cell line.

Supplementary Figure 6 Supplementary Figure 6. Time-course analysis of siRNA-mediated down regulation of GDF-15 in EHE cell line.

Supplementary Figure 7 Supplementary Figure 7. Volcano plot of genes differentially expressed between EHE cells transfected with GDF-15 siRNA or control (NEG) siRNA.

Supplementary Figure 8 Supplementary Figure 8. GDF-15 silencing did not impair the proliferative potential of EHE cells as well as their migration and invasion and ability to growth in vivo.

Supplementary Figure 9 Supplementary Figure 9. Representative images of H&E-stained EHE PDX tissue sections at the appropriate magnification to exhibit the different presence of sclerojalinosis (S) and necrotic (N) areas induced by Sirolimus at different doses.

Supplementary Figure 10 Supplementary Figure 10. GDF-15 down-regulation induced by sirolimus and/or ATF4/5 silencing.

Supplementary Table 1 Supplementary Table 1. Quantification of band intensities for blot reported in Figure 2D.

Supplementary Table 2 Supplementary Table 2. Quantification of band intensities for blot reported in Figure 3E.

Supplementary Table 3 Supplementary Table 3. Quantification of band intensities for blot reported in Figure 4C and E.

Supplementary Table 4 Supplementary Table 4. Drug schedules and tumor responses in the EHE PDX model.
