# Supplementary material for: GDF-15 Predicts Epithelioid Hemangioendothelioma Aggressiveness and Is Downregulated by Sirolimus through ATF4/ATF5 Suppression
Source: Clin Cancer Res. 2024 Sep 16;30(22):5122–37. doi: 10.1158/1078-0432.CCR-23-3991 (PMC11565171; doi:10.1158/1078-0432.CCR-23-3991)
Supplement: Supplementary Material 1 — Patient and human tumor characterization and prospective study. [file ccr-23-3991_supplementary_material_1_suppsm1.docx]

Supplementary materials - Summary

[SUPPLEMENTARY MATERIALS 2](#_Toc174115973)

[Patient and human tumor characterization 2](#_Toc174115974)

[Prospective study 2](#_Toc174115975)

# SUPPLEMENTARY MATERIALS

## Patient and human tumor characterization

A 63-year-old female patient with recurrent EHE in her left forearm was treated with surgery at the Sarcoma Service of INT following inadequate surgery performed 10 months earlier at another institution. EHE was diagnosed upon histomorphologic review and was confirmed with immunohistochemistry and FISH analysis. This patient did not receive any preoperative treatment. Surgery included a wide excision of the anterior compart of the right forearm. The tumor was 7 cm in its largest diameter. Microscopically, it was composed of spindle cells with diffuse cellular atypia, together with myxoid stroma and areas of sclero-jalinosis. IHC showed immunoreactivity for ERG (Abcam Cat# ab92513, RRID:AB_2630401), CD31 (Agilent Cat# M0823, RRID:AB_2114471), CAMTA1 (Novus Cat# NBP1-93620, RRID:AB_11038138), and p53 (DO7) (Agilent Cat# GA616, RRID:AB_2889978) and absence of CK AE1AE3 (Millipore Cat# MAB3412, RRID:AB_94853), EMA (Leica Biosystems Cat# NCL-EMA, RRID:AB_442087) and TFE3 (Cell Marque Cat# 354R-18, RRID:AB_3251504). Mib-1 (Ki-67 (Agilent Cat# GA626, RRID:AB_2687921)) was 25% and the tumor had a mitotic rate of 2/10HPF. FISH analyses (WWTR1 dual color break apart and WWTR1::CAMTA1 dual color dual fusion) showed an unbalanced translocation of WWTR1, with loss in the 3’ portion of WWTR1 and, consistently, a single WWTR1::CAMTA1 fusion. This patient presented with severe systemic symptoms, including cancer-related pain and developed systemic widespread disease progression including pleural effusions. The patient ultimately succumbed to the disease.

## Prospective study

This study is aimed at 1) describing the clinical presentation, natural history, and treatment outcomes of EHE patients; 2) evaluating circulating biomarkers; and 3) generating patient-derived preclinical models to assess the activity of anticancer agents and validate novel therapeutic targets. The study was started at INT and RMCC in May 2021. This study allows inclusion of i) newly diagnosed patients, naturally stable disease on follow-up; ii) patients with progressive disease requiring treatment; iii) patients with previously diagnosed EHE already on active surveillance; iv) patients on treatment; v) patients with localized EHE. Patients were classified as low- and high-risk based on disease behavior as follow: i) lower-risk, defined as absence of progressive over 12 months or evidence of progressive disease between 6 and 12 months and patient belonging to group D (provided that treatment was started in evidence on progressive disease); ii) higher-risk, defined as evidence of progressive disease within 6 months. Indeed, progressive disease was defined as follow: i) evidence of RECIST 1.1 progression; ii) any increase in size of the known lesions (even not meeting RECIST 1.1 definition of progression) in association with worsening of at least two tumor related symptoms among tumor-related pain, fever, weight loss, asthenia; iii) new appearance or any worsening of serosal effusion or involvement in association with worsening of at least two tumor-related symptoms among tumor related pain, fever, weight loss, asthenia. Worsening of tumor-related pain was defined as an increase in tumor-related pain of 2 points on NRS from 0 to 10 of at least 2-week duration or new onset of tumor-related pain of at least 3/10 of 1 week duration.

All study procedures were performed in agreement with the Declaration of Helsinki. All patients gave their written informed consent to participate in the study. Approval from the INT and RMCC ethic committees was obtained. The study was funded by a research grant from EHE UK and US,

In addition, for comparison, by protocol blood samples were also collected from 32 healthy patients having the same sex and age characteristics.
