# Supplementary material for: GDF-15 Predicts Epithelioid Hemangioendothelioma Aggressiveness and Is Downregulated by Sirolimus through ATF4/ATF5 Suppression
Source: Clin Cancer Res. 2024 Sep 16;30(22):5122–37. doi: 10.1158/1078-0432.CCR-23-3991 (PMC11565171; doi:10.1158/1078-0432.CCR-23-3991)
Supplement: Supplementary Figure 1 — Representative growth curves of the EHE PDX at different passages in mice. [file ccr-23-3991_supplementary_figure_1_suppsf1.pptx]

## Slide 1
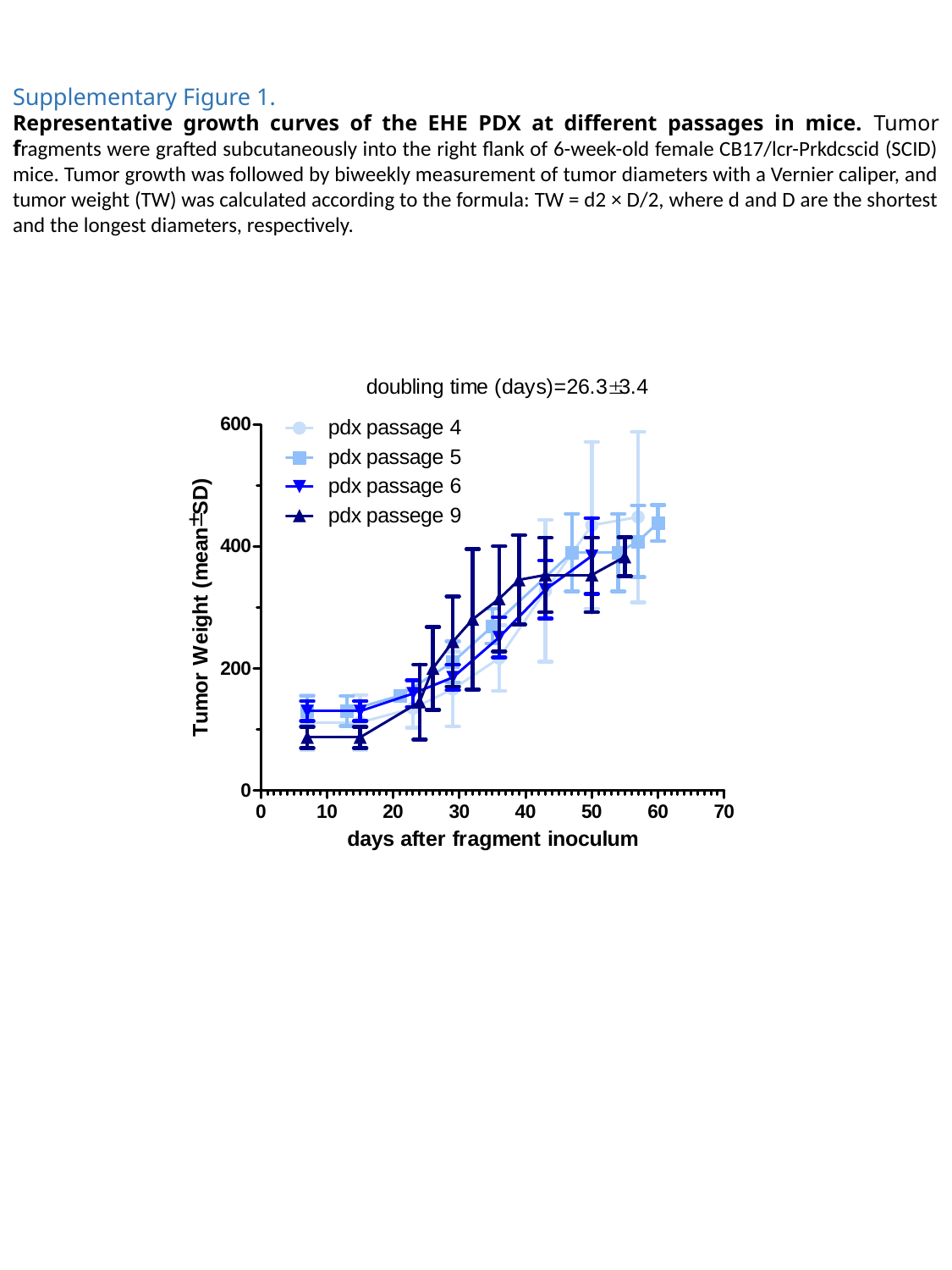

Supplementary Figure 1.
Representative growth curves of the EHE PDX at different passages in mice. Tumor fragments were grafted subcutaneously into the right flank of 6-week-old female CB17/lcr-Prkdcscid (SCID) mice. Tumor growth was followed by biweekly measurement of tumor diameters with a Vernier caliper, and tumor weight (TW) was calculated according to the formula: TW = d2 × D/2, where d and D are the shortest and the longest diameters, respectively.
