# Supplementary material for: GDF-15 Predicts Epithelioid Hemangioendothelioma Aggressiveness and Is Downregulated by Sirolimus through ATF4/ATF5 Suppression
Source: Clin Cancer Res. 2024 Sep 16;30(22):5122–37. doi: 10.1158/1078-0432.CCR-23-3991 (PMC11565171; doi:10.1158/1078-0432.CCR-23-3991)
Supplement: Supplementary Figure 2 — CDKN2A loss in EHE clinical tumor and PDX. [file ccr-23-3991_supplementary_figure_2_suppsf2.pptx]

## Slide 1
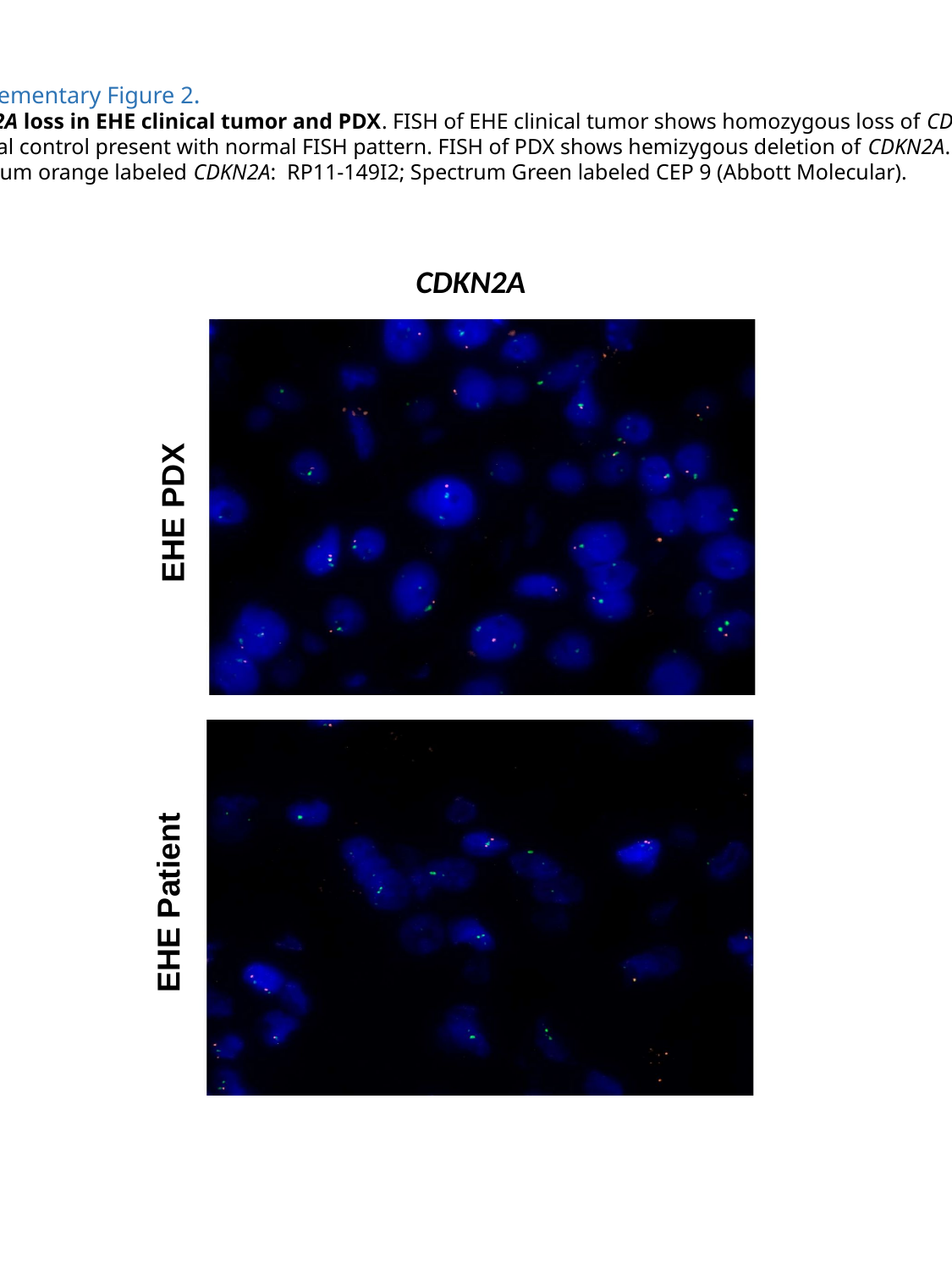

Supplementary Figure 2.
CDKN2A loss in EHE clinical tumor and PDX. FISH of EHE clinical tumor shows homozygous loss of CDKN2A;
internal control present with normal FISH pattern. FISH of PDX shows hemizygous deletion of CDKN2A.
Spectrum orange labeled CDKN2A: RP11-149I2; Spectrum Green labeled CEP 9 (Abbott Molecular).
CDKN2A
EHE PDX
EHE Patient
