# Supplementary material for: GDF-15 Predicts Epithelioid Hemangioendothelioma Aggressiveness and Is Downregulated by Sirolimus through ATF4/ATF5 Suppression
Source: Clin Cancer Res. 2024 Sep 16;30(22):5122–37. doi: 10.1158/1078-0432.CCR-23-3991 (PMC11565171; doi:10.1158/1078-0432.CCR-23-3991)
Supplement: Supplementary Figure 3 — Chromosomal architecture of the PDX compared to the clinical sample in chromosomes 7 and 11. [file ccr-23-3991_supplementary_figure_3_suppsf3.pptx]

## Slide 1
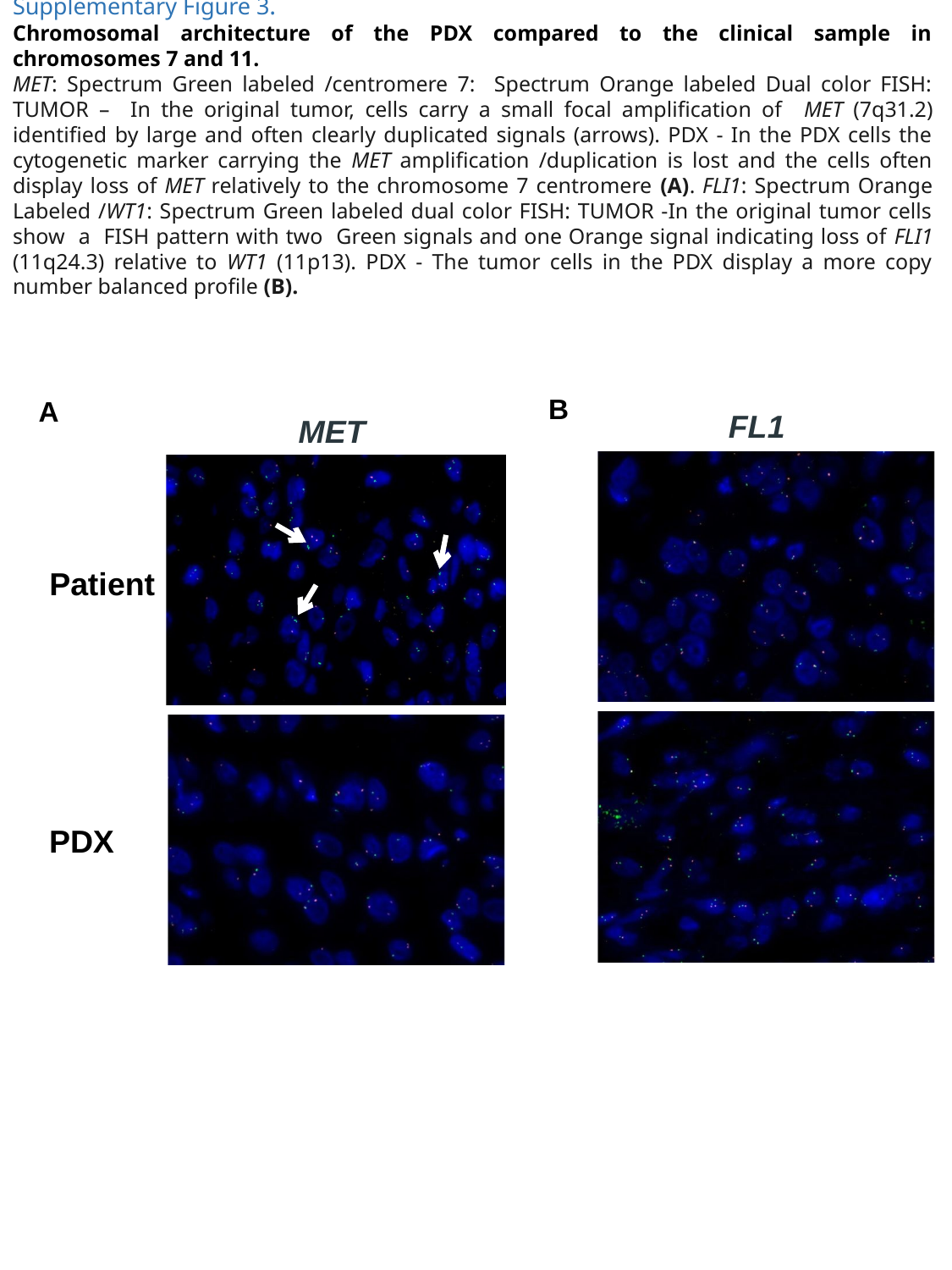

Supplementary Figure 3.
Chromosomal architecture of the PDX compared to the clinical sample in chromosomes 7 and 11.
MET: Spectrum Green labeled /centromere 7: Spectrum Orange labeled Dual color FISH: TUMOR – In the original tumor, cells carry a small focal amplification of MET (7q31.2) identified by large and often clearly duplicated signals (arrows). PDX - In the PDX cells the cytogenetic marker carrying the MET amplification /duplication is lost and the cells often display loss of MET relatively to the chromosome 7 centromere (A). FLI1: Spectrum Orange Labeled /WT1: Spectrum Green labeled dual color FISH: TUMOR -In the original tumor cells show a FISH pattern with two Green signals and one Orange signal indicating loss of FLI1 (11q24.3) relative to WT1 (11p13). PDX - The tumor cells in the PDX display a more copy number balanced profile (B).
B
A
FL1
MET
Patient
PDX
