# Supplementary material for: GDF-15 Predicts Epithelioid Hemangioendothelioma Aggressiveness and Is Downregulated by Sirolimus through ATF4/ATF5 Suppression
Source: Clin Cancer Res. 2024 Sep 16;30(22):5122–37. doi: 10.1158/1078-0432.CCR-23-3991 (PMC11565171; doi:10.1158/1078-0432.CCR-23-3991)
Supplement: Supplementary Figure 4 — Transcriptomic profiles of EHE clinical tumor and PDX. [file ccr-23-3991_supplementary_figure_4_suppsf4.pptx]

## Slide 1
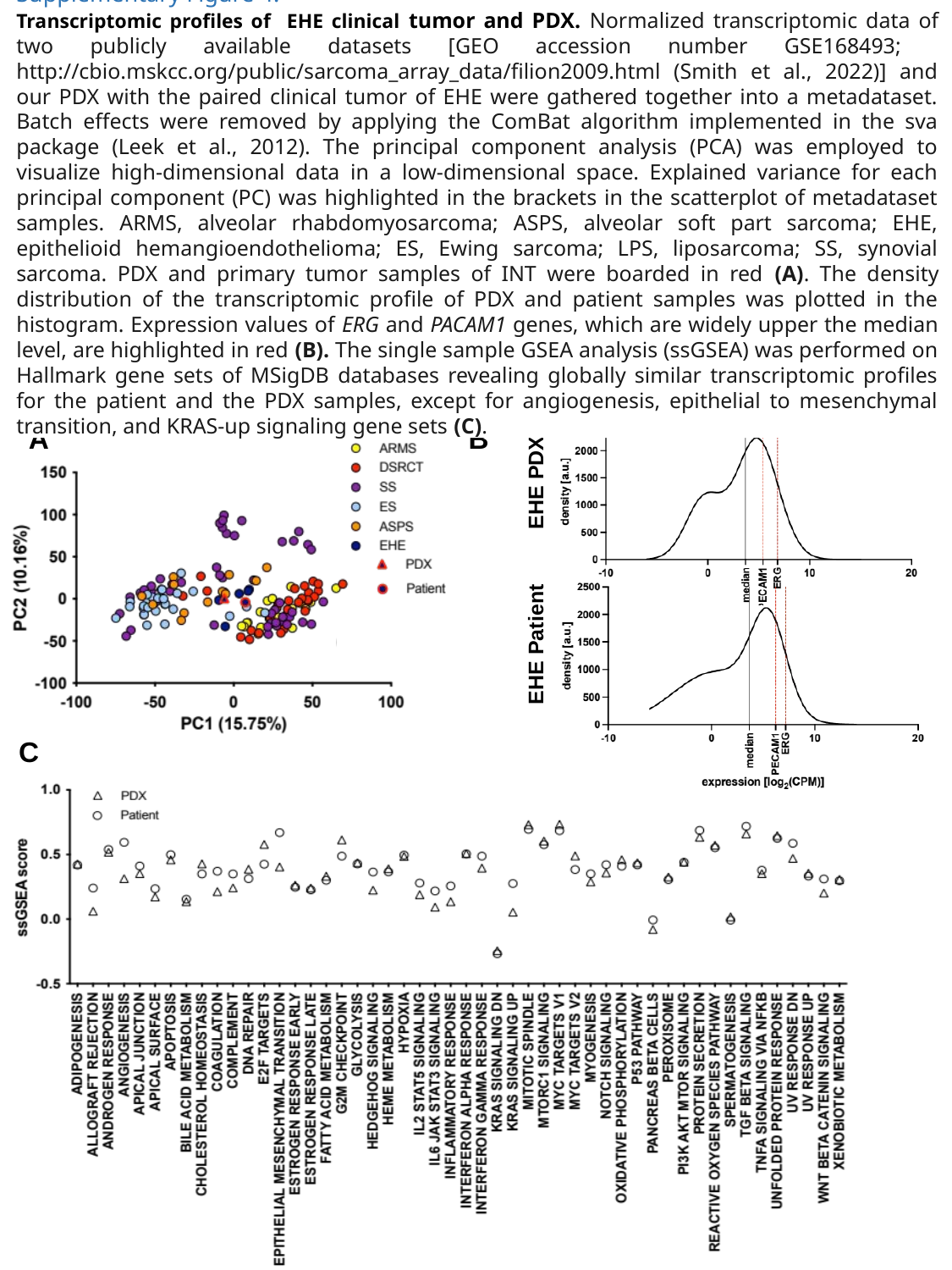

Supplementary Figure 4.
Transcriptomic profiles of EHE clinical tumor and PDX. Normalized transcriptomic data of two publicly available datasets [GEO accession number GSE168493; http://cbio.mskcc.org/public/sarcoma_array_data/filion2009.html (Smith et al., 2022)] and our PDX with the paired clinical tumor of EHE were gathered together into a metadataset. Batch effects were removed by applying the ComBat algorithm implemented in the sva package (Leek et al., 2012). The principal component analysis (PCA) was employed to visualize high-dimensional data in a low-dimensional space. Explained variance for each principal component (PC) was highlighted in the brackets in the scatterplot of metadataset samples. ARMS, alveolar rhabdomyosarcoma; ASPS, alveolar soft part sarcoma; EHE, epithelioid hemangioendothelioma; ES, Ewing sarcoma; LPS, liposarcoma; SS, synovial sarcoma. PDX and primary tumor samples of INT were boarded in red (A). The density distribution of the transcriptomic profile of PDX and patient samples was plotted in the histogram. Expression values of ERG and PACAM1 genes, which are widely upper the median level, are highlighted in red (B). The single sample GSEA analysis (ssGSEA) was performed on Hallmark gene sets of MSigDB databases revealing globally similar transcriptomic profiles for the patient and the PDX samples, except for angiogenesis, epithelial to mesenchymal transition, and KRAS-up signaling gene sets (C).
A
B
EHE PDX
EHE Patient
C
