# Supplementary material for: GDF-15 Predicts Epithelioid Hemangioendothelioma Aggressiveness and Is Downregulated by Sirolimus through ATF4/ATF5 Suppression
Source: Clin Cancer Res. 2024 Sep 16;30(22):5122–37. doi: 10.1158/1078-0432.CCR-23-3991 (PMC11565171; doi:10.1158/1078-0432.CCR-23-3991)
Supplement: Supplementary Figure 5 — Effect of different siRNA-mediated down-regulation of GDF-15 in EHE cell line. [file ccr-23-3991_supplementary_figure_5_suppsf5.pptx]

## Slide 1
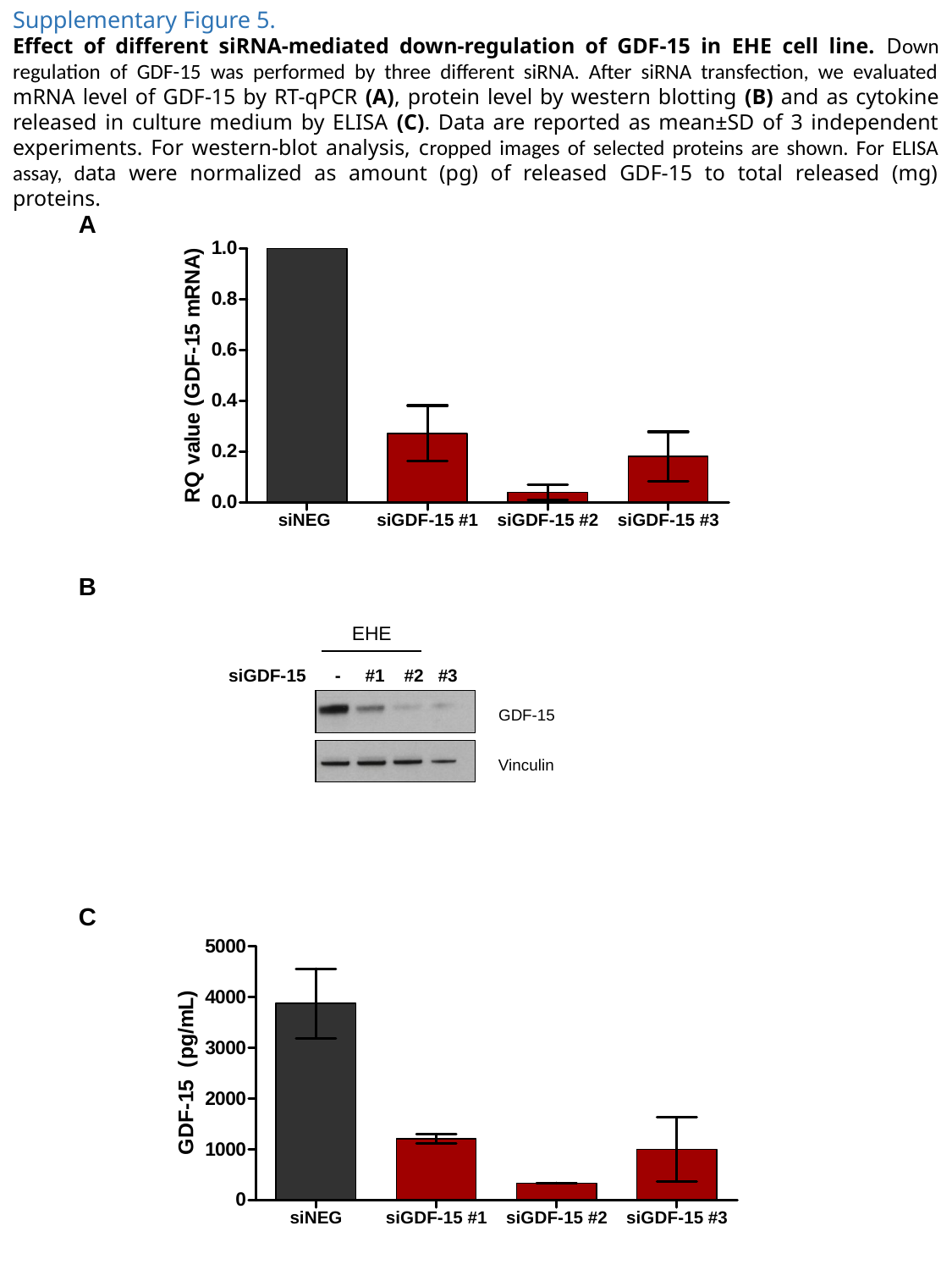

Supplementary Figure 5.
Effect of different siRNA-mediated down-regulation of GDF-15 in EHE cell line. Down regulation of GDF-15 was performed by three different siRNA. After siRNA transfection, we evaluated mRNA level of GDF-15 by RT-qPCR (A), protein level by western blotting (B) and as cytokine released in culture medium by ELISA (C). Data are reported as mean±SD of 3 independent experiments. For western-blot analysis, cropped images of selected proteins are shown. For ELISA assay, data were normalized as amount (pg) of released GDF-15 to total released (mg) proteins.
A
B
EHE
siGDF-15 - #1 #2 #3
GDF-15
Vinculin
C
