# Supplementary material for: GDF-15 Predicts Epithelioid Hemangioendothelioma Aggressiveness and Is Downregulated by Sirolimus through ATF4/ATF5 Suppression
Source: Clin Cancer Res. 2024 Sep 16;30(22):5122–37. doi: 10.1158/1078-0432.CCR-23-3991 (PMC11565171; doi:10.1158/1078-0432.CCR-23-3991)
Supplement: Supplementary Figure 6 — Time-course analysis of siRNA-mediated down regulation of GDF-15 in EHE cell line. [file ccr-23-3991_supplementary_figure_6_suppsf6.pptx]

## Slide 1
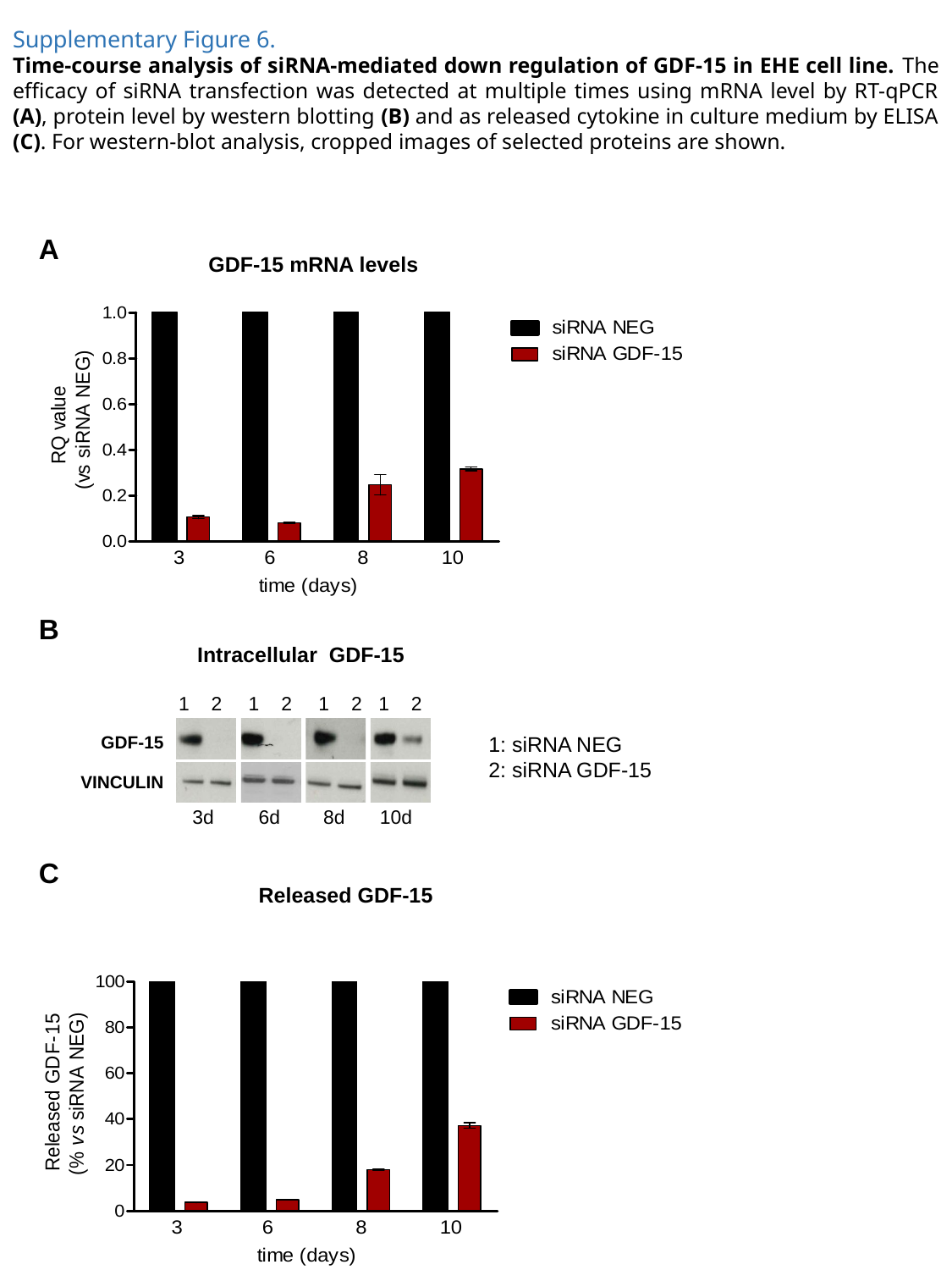

Supplementary Figure 6.
Time-course analysis of siRNA-mediated down regulation of GDF-15 in EHE cell line. The efficacy of siRNA transfection was detected at multiple times using mRNA level by RT-qPCR (A), protein level by western blotting (B) and as released cytokine in culture medium by ELISA (C). For western-blot analysis, cropped images of selected proteins are shown.
A
GDF-15 mRNA levels
B
Intracellular GDF-15
1 2
1 2
1 2
1 2
GDF-15
1: siRNA NEG
2: siRNA GDF-15
VINCULIN
3d
6d
8d
10d
C
Released GDF-15
