# Supplementary material for: GDF-15 Predicts Epithelioid Hemangioendothelioma Aggressiveness and Is Downregulated by Sirolimus through ATF4/ATF5 Suppression
Source: Clin Cancer Res. 2024 Sep 16;30(22):5122–37. doi: 10.1158/1078-0432.CCR-23-3991 (PMC11565171; doi:10.1158/1078-0432.CCR-23-3991)
Supplement: Supplementary Figure 7 — Volcano plot of genes differentially expressed between EHE cells transfected with GDF-15 siRNA or control (NEG) siRNA. [file ccr-23-3991_supplementary_figure_7_suppsf7.pptx]

## Slide 1
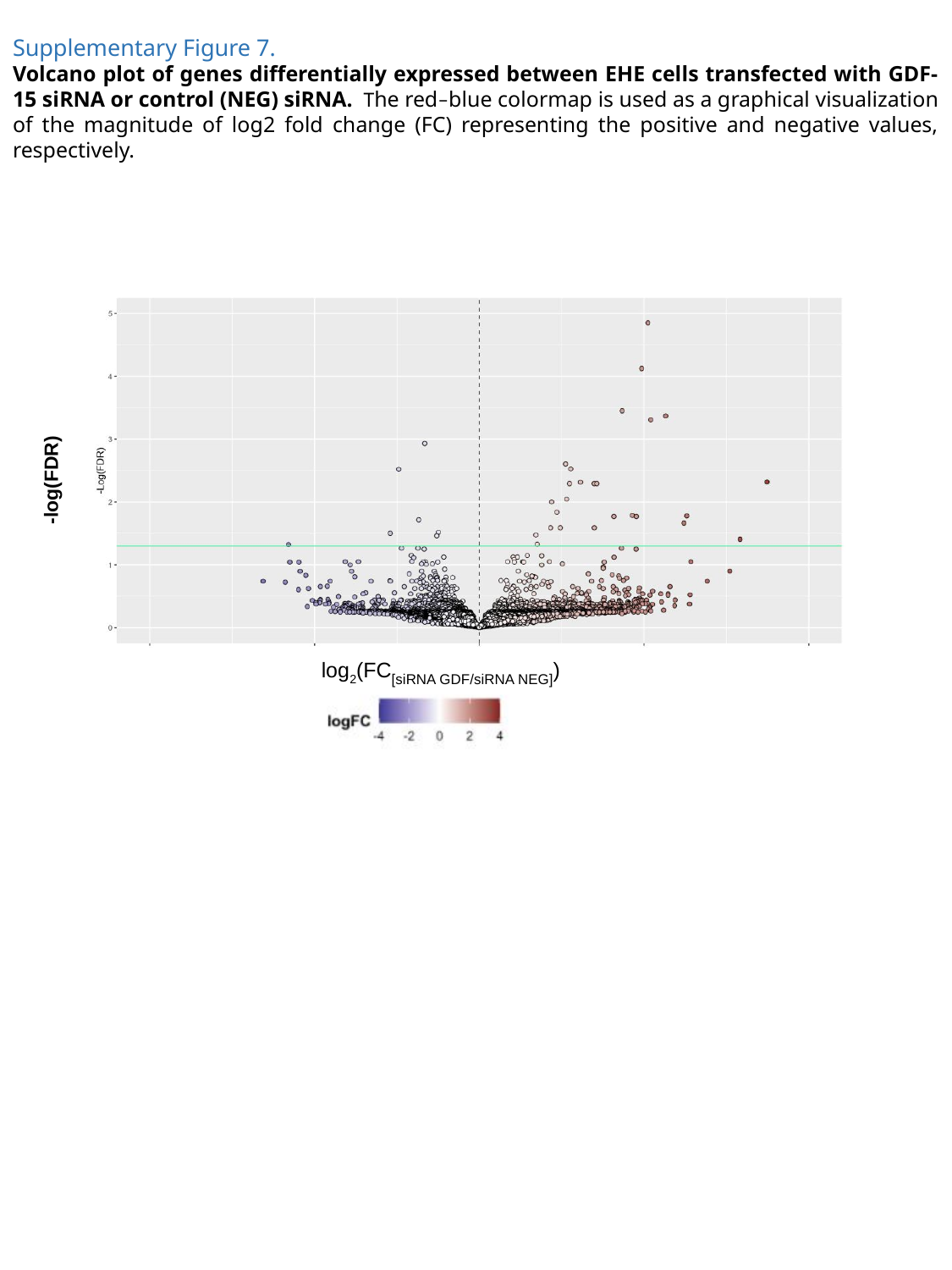

Supplementary Figure 7.
Volcano plot of genes differentially expressed between EHE cells transfected with GDF-15 siRNA or control (NEG) siRNA. The red–blue colormap is used as a graphical visualization of the magnitude of log2 fold change (FC) representing the positive and negative values, respectively.
-log(FDR)
log2(FC[siRNA GDF/siRNA NEG])
