# Supplementary material for: GDF-15 Predicts Epithelioid Hemangioendothelioma Aggressiveness and Is Downregulated by Sirolimus through ATF4/ATF5 Suppression
Source: Clin Cancer Res. 2024 Sep 16;30(22):5122–37. doi: 10.1158/1078-0432.CCR-23-3991 (PMC11565171; doi:10.1158/1078-0432.CCR-23-3991)
Supplement: Supplementary Figure 8 — GDF-15 silencing did not impair the proliferative potential of EHE cells as well as their migration and invasion and ability to growth in vivo. [file ccr-23-3991_supplementary_figure_8_suppsf8.pptx]

## Slide 1
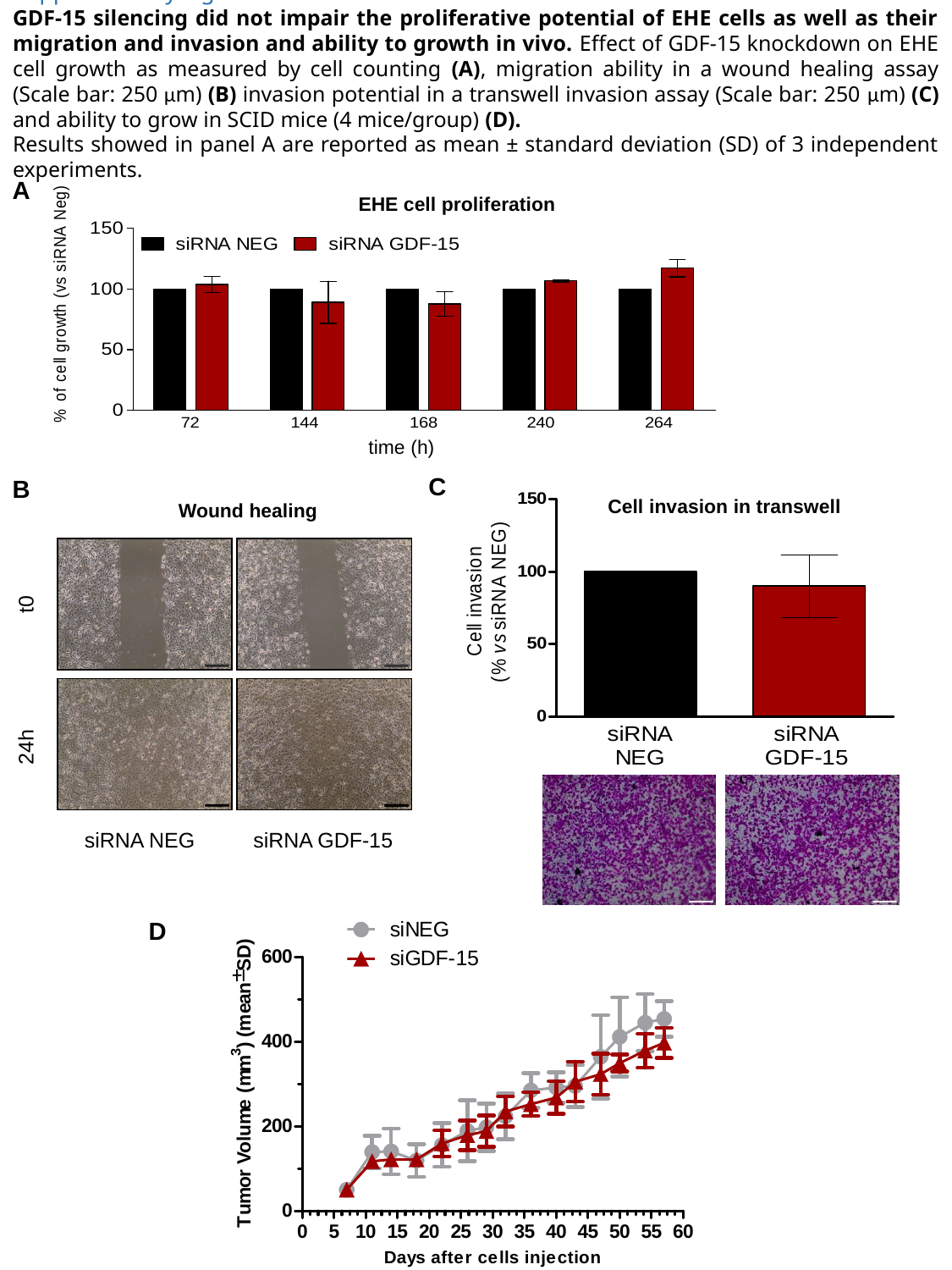

Supplementary Figure 8.
GDF-15 silencing did not impair the proliferative potential of EHE cells as well as their migration and invasion and ability to growth in vivo. Effect of GDF-15 knockdown on EHE cell growth as measured by cell counting (A), migration ability in a wound healing assay (Scale bar: 250 µm) (B) invasion potential in a transwell invasion assay (Scale bar: 250 µm) (C) and ability to grow in SCID mice (4 mice/group) (D).
Results showed in panel A are reported as mean ± standard deviation (SD) of 3 independent experiments.
A
EHE cell proliferation
time (h)
C
B
Cell invasion in transwell
Wound healing
t0
24h
siRNA NEG
siRNA GDF-15
D
