# Supplementary material for: GDF-15 Predicts Epithelioid Hemangioendothelioma Aggressiveness and Is Downregulated by Sirolimus through ATF4/ATF5 Suppression
Source: Clin Cancer Res. 2024 Sep 16;30(22):5122–37. doi: 10.1158/1078-0432.CCR-23-3991 (PMC11565171; doi:10.1158/1078-0432.CCR-23-3991)
Supplement: Supplementary Figure 9 — Representative images of H&E-stained EHE PDX tissue sections at the appropriate magnification to exhibit the different presence of sclerojalinosis (S) and necrotic (N) areas induced by Sirolimus at different doses. [file ccr-23-3991_supplementary_figure_9_suppsf9.pptx]

## Slide 1
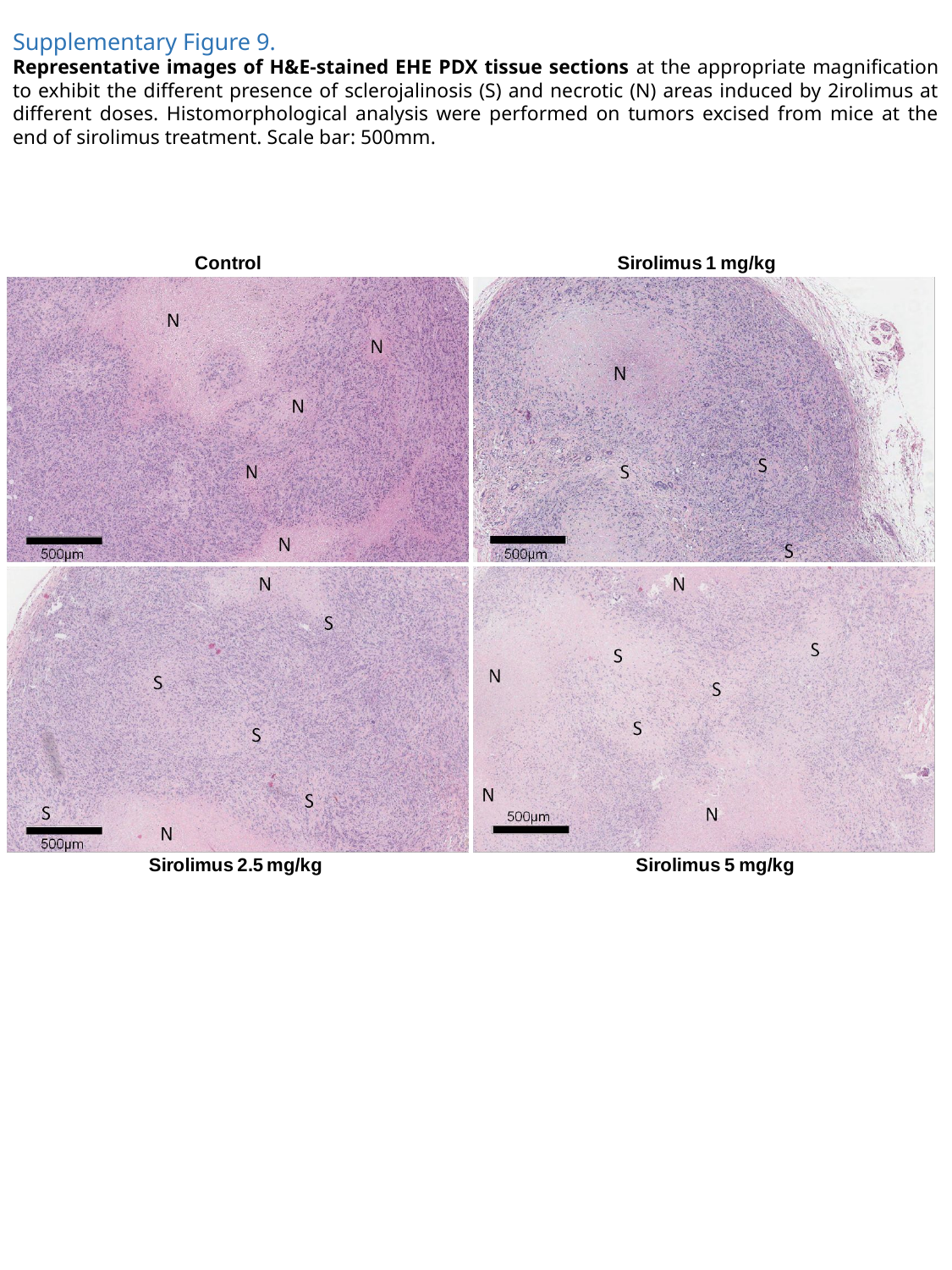

Supplementary Figure 9.
Representative images of H&E-stained EHE PDX tissue sections at the appropriate magnification to exhibit the different presence of sclerojalinosis (S) and necrotic (N) areas induced by 2irolimus at different doses. Histomorphological analysis were performed on tumors excised from mice at the end of sirolimus treatment. Scale bar: 500mm.
