# Supplementary material for: GDF-15 Predicts Epithelioid Hemangioendothelioma Aggressiveness and Is Downregulated by Sirolimus through ATF4/ATF5 Suppression
Source: Clin Cancer Res. 2024 Sep 16;30(22):5122–37. doi: 10.1158/1078-0432.CCR-23-3991 (PMC11565171; doi:10.1158/1078-0432.CCR-23-3991)
Supplement: Supplementary Figure 10 — GDF-15 down-regulation induced by sirolimus and/or ATF4/5 silencing. [file ccr-23-3991_supplementary_figure_10_suppsf10.pptx]

## Slide 1
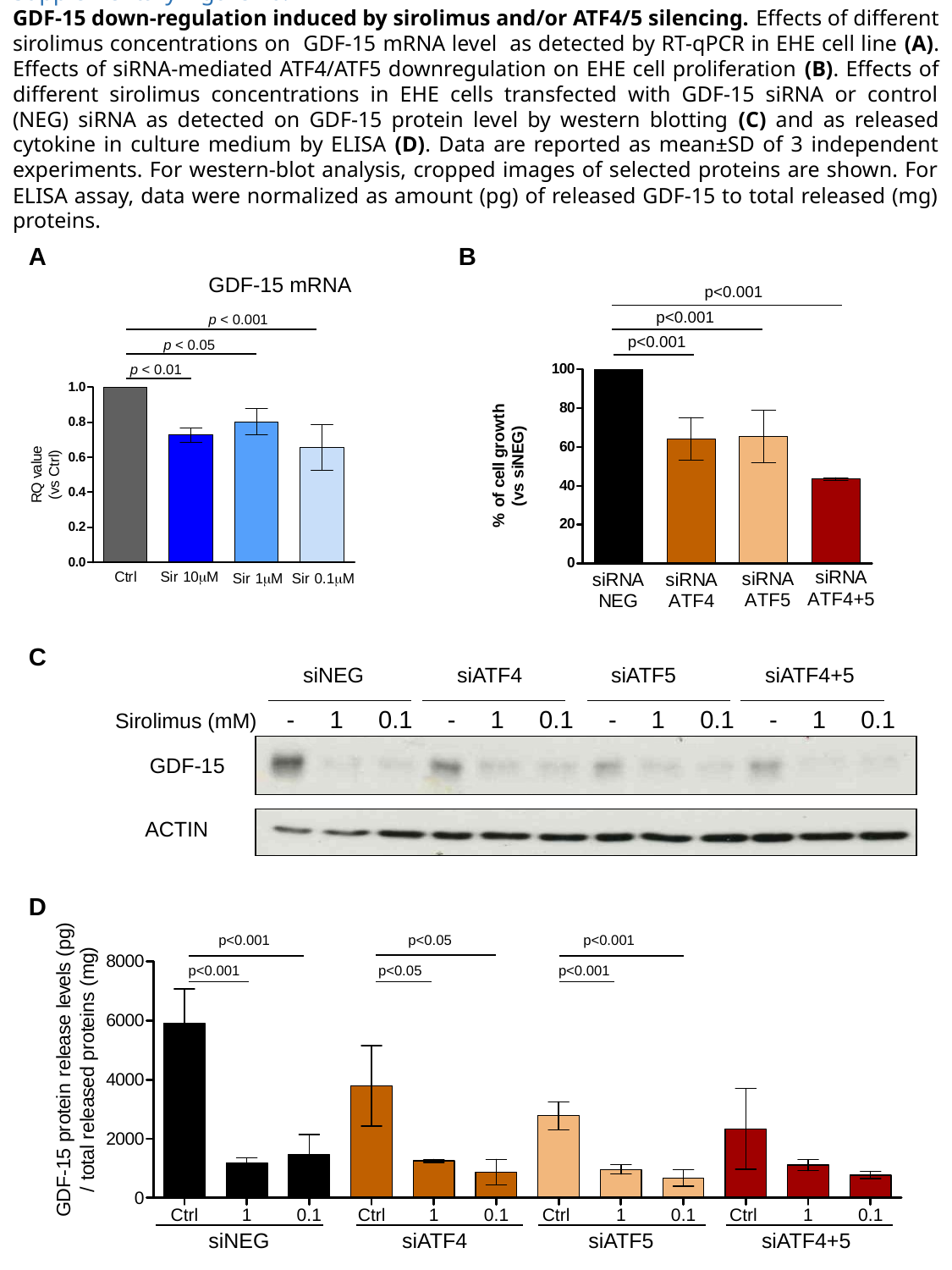

Supplementary Figure 10.
GDF-15 down-regulation induced by sirolimus and/or ATF4/5 silencing. Effects of different sirolimus concentrations on GDF-15 mRNA level as detected by RT-qPCR in EHE cell line (A). Effects of siRNA-mediated ATF4/ATF5 downregulation on EHE cell proliferation (B). Effects of different sirolimus concentrations in EHE cells transfected with GDF-15 siRNA or control (NEG) siRNA as detected on GDF-15 protein level by western blotting (C) and as released cytokine in culture medium by ELISA (D). Data are reported as mean±SD of 3 independent experiments. For western-blot analysis, cropped images of selected proteins are shown. For ELISA assay, data were normalized as amount (pg) of released GDF-15 to total released (mg) proteins.
A
B
GDF-15 mRNA
p<0.001
p<0.001
p < 0.001
p<0.001
p < 0.05
p < 0.01
C
siNEG
siATF4
siATF5
siATF4+5
 Sirolimus (mM) - 1 0.1 - 1 0.1 - 1 0.1 - 1 0.1
GDF-15
ACTIN
D
p<0.001
p<0.05
p<0.001
p<0.001
p<0.05
p<0.001
siNEG
siATF4
siATF5
siATF4+5
