# Supplementary material for: GDF-15 Predicts Epithelioid Hemangioendothelioma Aggressiveness and Is Downregulated by Sirolimus through ATF4/ATF5 Suppression
Source: Clin Cancer Res. 2024 Sep 16;30(22):5122–37. doi: 10.1158/1078-0432.CCR-23-3991 (PMC11565171; doi:10.1158/1078-0432.CCR-23-3991)
Supplement: Supplementary Table 1 — Quantification of band intensities for blot reported in Figure 2D. [file ccr-23-3991_supplementary_table_1_suppst1.pptx]

## Slide 1
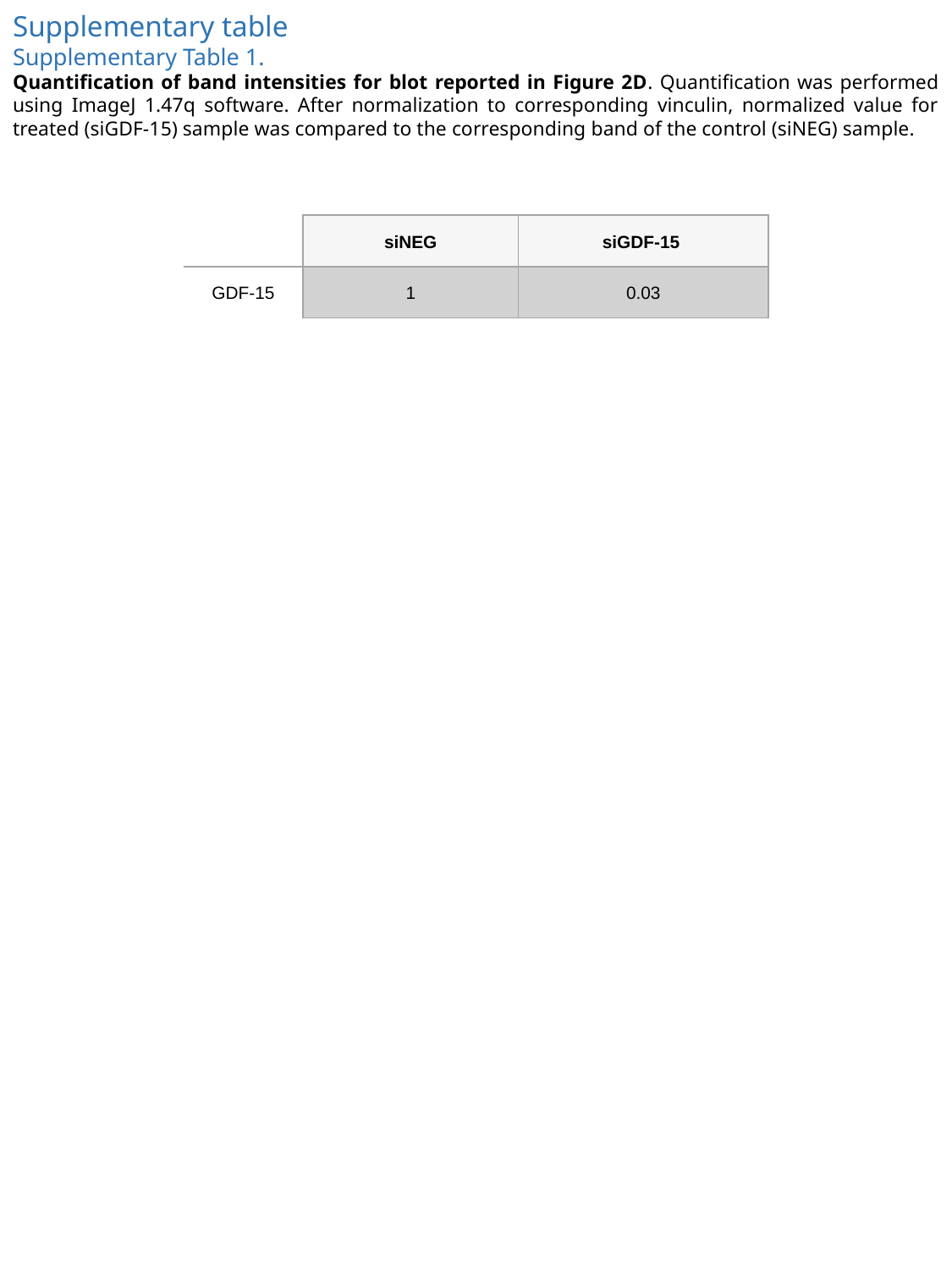

Supplementary table
Supplementary Table 1.
Quantification of band intensities for blot reported in Figure 2D. Quantification was performed using ImageJ 1.47q software. After normalization to corresponding vinculin, normalized value for treated (siGDF-15) sample was compared to the corresponding band of the control (siNEG) sample.
| | siNEG | siGDF-15 |
| --- | --- | --- |
| GDF-15 | 1 | 0.03 |
