# Supplementary material for: GDF-15 Predicts Epithelioid Hemangioendothelioma Aggressiveness and Is Downregulated by Sirolimus through ATF4/ATF5 Suppression
Source: Clin Cancer Res. 2024 Sep 16;30(22):5122–37. doi: 10.1158/1078-0432.CCR-23-3991 (PMC11565171; doi:10.1158/1078-0432.CCR-23-3991)
Supplement: Supplementary Table 3 — Quantification of band intensities for blot reported in Figure 4C and E. [file ccr-23-3991_supplementary_table_3_suppst3.pptx]

## Slide 1
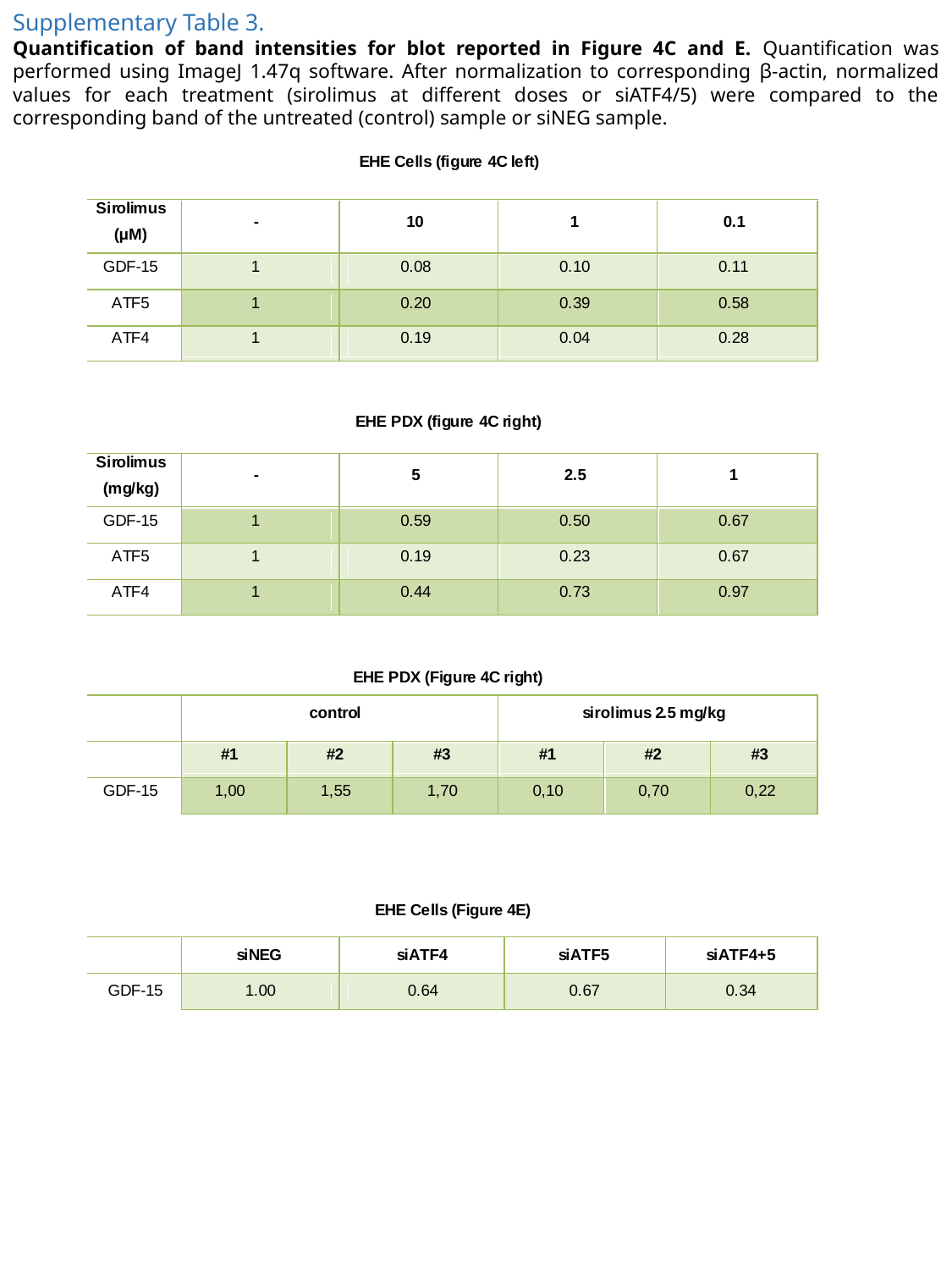

Supplementary Table 3.
Quantification of band intensities for blot reported in Figure 4C and E. Quantification was performed using ImageJ 1.47q software. After normalization to corresponding β-actin, normalized values for each treatment (sirolimus at different doses or siATF4/5) were compared to the corresponding band of the untreated (control) sample or siNEG sample.
