# Supplementary figures and images for: GDF-15 Predicts Epithelioid Hemangioendothelioma Aggressiveness and Is Downregulated by Sirolimus through ATF4/ATF5 Suppression
Source: Clin Cancer Res. 2024 Sep 16;30(22):5122–37. doi: 10.1158/1078-0432.CCR-23-3991 (PMC11565171; doi:10.1158/1078-0432.CCR-23-3991)

## Slide 1
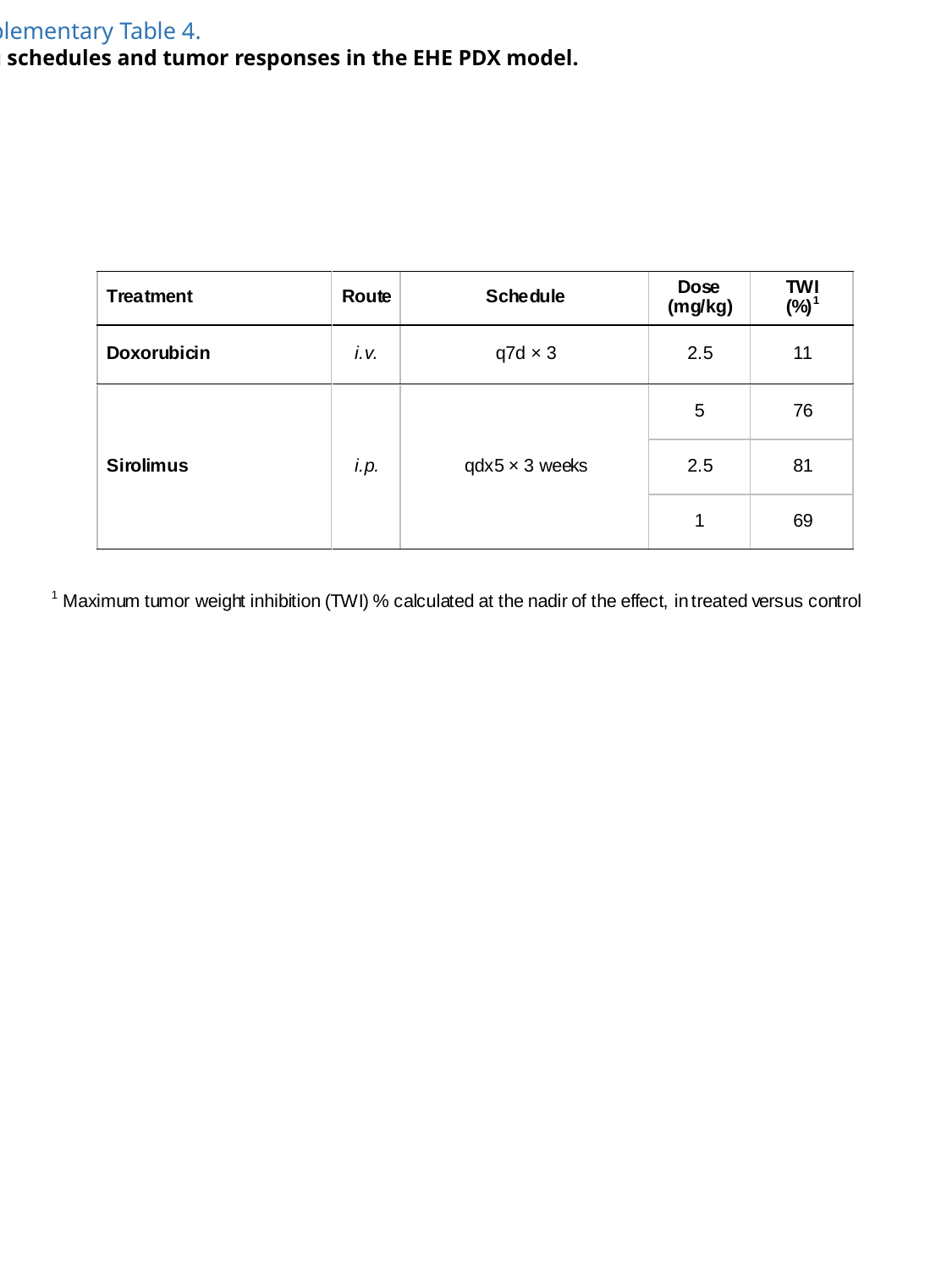

Supplementary Table 4.
Drug schedules and tumor responses in the EHE PDX model.

Supplement: Supplementary Table 4 — Drug schedules and tumor responses in the EHE PDX model. [file ccr-23-3991_supplementary_table_4_suppst4.pptx]
